# The P2Y_11_ receptor of human M2 macrophages activates canonical and IL-1 receptor signaling to translate the extracellular danger signal ATP into anti-inflammatory and pro-angiogenic responses

**DOI:** 10.1007/s00018-022-04548-z

**Published:** 2022-09-15

**Authors:** Dominik Klaver, Hubert Gander, Gabriele Dobler, Andrea Rahm, Martin Thurnher

**Affiliations:** Immunotherapy Unit, Department of Urology, Medical University of Innsbruck, Innrain 66a, 6020 Innsbruck, Austria

**Keywords:** Macrophage polarization, Purinergic signaling, Homeostasis, NanoString

## Abstract

The cytoprotective ATP receptor P2Y_11_ is upregulated during M2 macrophage differentiation and contributes to the anti-inflammatory properties of this macrophage subset. Here, we studied P2Y_11_-induced reprogramming of human M2 macrophages at the level of mRNA and protein expression. Upregulation of IL-1 receptor (IL-1R) and its known downstream effectors VEGF, CCL20 and SOCS3 as well as downregulation of the ATP-degrading ecto-ATPase CD39 emerged as hallmarks of P2Y_11_ activation. The anti-inflammatory signature of the P2Y_11_ transcriptome was further characterized by the downregulation of P2RX7, toll-like receptors and inflammasome components. P2Y_11_-induced IL-1R upregulation formed the basis for reinforced IL-1 responsiveness of activated M2 macrophages, as IL-1α and IL-1ß each enhanced P2Y_11_-induced secretion of VEGF and CCL20 as well as the previously reported shedding of soluble tumor necrosis factor receptor 2 (sTNFR2). Raising intracellular cyclic AMP (cAMP) in M2 macrophages through phosphodiesterase 4 inhibition enhanced P2Y_11_-driven responses. The cAMP-binding effector, exchange protein activated by cAMP 1 (Epac1), which is known to induce SOCS3, differentially regulated the P2Y_11_/IL-1R response because pharmacological Epac1 inhibition enhanced sTNFR2 and CCL20 release, but had no effect on VEGF secretion. In addition to cAMP, calcium and protein kinase C participated in P2Y_11_ signaling. Our study reveals how P2Y_11_ harnesses canonical and IL-1R signaling to promote an anti-inflammatory and pro-angiogenic switch of human M2 macrophages, which may be controlled in part by an Epac1-SOCS3 axis.

**Supplementary Information:**

The online version contains supplementary material available at 10.1007/s00018-022-04548-z.

## Introduction

The concentration of adenosine triphosphate (ATP) in interstitial fluid of undisturbed tissues remains in the nanomolar range. However, stressed cells release ATP in a controlled or lytic manner, raising the levels of extracellular ATP to low or even high micromolar concentrations. For instance during infection, microbial products stimulate toll-like receptors (TLRs) to induce enhanced ATP release [1–3]. Extracellular ATP can now act as a damage-associated molecular pattern (DAMP) and cause the coordinate activation of ATP-responsive P2 purinergic receptors on immune cells [4, 5], which translate the ATP alarm into either pro-inflammatory, cytolytic (e.g. P2X_7_) or immunomodulatory, cytoprotective responses (e.g. P2Y_11_).

P2Y_11_ is an atypical representative of the P2Y family of G protein-coupled receptors (GPCRs), as it can couple to both, G_q_ and G_s_ proteins, and does not exist in rodents [6, 7]. Among immune cells, P2Y_11_ has been studied mainly in dendritic cells, macrophages and in T cells [8–11]. We, and others, have recently shown that P2Y_11_ mRNA and protein expression substantially increase during monocyte-to-macrophage differentiation [8, 12].

The lack of rodent models and the limited availability of P2Y_11_-specific research tools has long slowed down the systematic examination of P2Y_11_ signaling [13, 14]. In a recent study, we used transcriptome profiling of recombinant P2Y_11_ expressed in a P2R-deficient astrocytoma cell line to examine P2Y_11_ downstream signaling [15]. We found that P2Y_11_ promoted the release (shedding) of soluble TNF receptors through TNF-α converting enzyme (TACE), also known as ADAM17 (a disintegrin and metalloprotease 17), both in astrocytoma cells (sTNFR1) and in human M2 macrophages (sTNFR2). Moreover, we found that P2Y_11_ blocks TLR4-driven TNF-α secretion in M2 macrophages, altogether establishing P2Y_11_ as a target for anti-inflammatory strategies [15].

G_q_ protein coupling of P2Y_11_ results in phospholipase Cß-catalyzed production of second messengers, which mobilize Ca^2+^ (via inositol trisphosphate, IP_3_) and activate protein kinase C (via diacylglycerol, DAG) [16]. By coupling to G_s_, P2Y_11_ can activate adenylyl cyclase (AC) and thus raise the intracellular levels of cAMP, which has potent anti-inflammatory activity [17]. Accordingly, we previously found that inhibition of cAMP degradation by blockade of PDE4 further enhanced the P2Y_11_-driven release of sTNFR2 from macrophages [15]. Intracellular cAMP acts via at least three mechanisms to modulate immune responses [17]: protein kinase A (PKA), also known as cAMP-dependent protein kinase, the two isoforms of exchange protein directly activated by cAMP, Epac1 and Epac2, as well as cyclic nucleotide-gated (CNG) ion channels in distinct cell types.

In the present work, we wanted to examine P2Y_11_ canonical and IL-1R signaling as well as the downstream effects that determine the functional phenotype of human M2 macrophages. We have established the first transcriptional profile of native P2Y_11_ signaling in its natural environment, also taking into account the levels of intracellular cyclic AMP. We not only provide evidence for P2Y_11_/IL-1R crosstalk in human M2 macrophages but also identify several novel P2Y_11_ target genes. In addition, the cAMP effector Epac1, a well-established inducer of suppressor of cytokine signaling 3 (SOCS3) [18, 19], turns out to be a regulator of P2Y_11_/IL-1R signaling. Collectively, the observed signature of response is consistent with a P2Y_11_-driven polarization of anti-inflammatory and pro-angiogenic M2 macrophages.

## Materials and methods

### Reagents

The slowly hydrolyzable ATP analog ATPγS [7, 20] (Sigma Aldrich, St. Louis, MO, USA) as well as the suramin analog NF340 [7, 10, 13, 21] (Santa Cruz, Dallas, TX, USA) served as P2Y_11_ receptor agonist (20 µM) and antagonist (20 µM), respectively. Further reagents utilized in this study include the PDE4-selective inhibitor rolipram (10 µM) (Sigma-Aldrich), BAPTA-AM (10 µM) (Sigma-Aldrich), calphostin C (250 nM) (Tocris), recombinant IL-1α and IL-1β (0.5–2 ng·ml^−1^) (R&D Systems, Minneapolis, MN, USA), the TACE/ADAM17 inhibitor TAPI-1 (20 µM) (Tocris), the selective inhibitor of Epac1 (R)-CE3F4 (20 µM) (Tocris), and the humanized anti-VEGF monoclonal antibody bevacizumab (0.5 µg ml^−1^) (Selleckchem, Houston, TX, USA).

### Isolation of human monocytes and generation of M2 macrophages

Buffy coats from randomly selected anonymous donors were provided by the Central Institute for Blood Transfusion (Innsbruck, Austria) after written informed consent. Inclusion of healthy donors in the present study was approved by the local Institutional Review board (ethical committee number: 1087/2018).

PBMCs were isolated from these buffy coats by density gradient centrifugation (Lymphoprep; Stem Cell Technologies, Vancouver, Canada). Subsequently, monocytes were isolated from PBMCs by positive selection using CD14 microbeads (human; 130-050-201, Miltenyi Biotec, Bergisch Gladbach, Germany) and LS columns (130-042-401, Miltenyi Biotec). Freshly isolated monocytes were differentiated toward M2 macrophages for 6 days in RPMI1640 (Lonza, Basel, Switzerland) supplemented with 20% FBS (HyClone, Logan, UT, USA), 1% GlutaMAX (100×; Gibco/Thermo Fisher Scientific, Waltham, MA, USA), 10 mM HEPES (1 M), 1 mM sodium pyruvate (100 mM), 1% nonessential amino acid mixture (NEAA; 100×) and 1% Pen/Strep (10.000 U ml^−1^; all from Lonza) in the presence of 50 ng·ml^−1^ macrophage colony-stimulating factor (M-CSF; Miltenyi Biotec). Fresh medium containing 50 ng·ml^−1^ M-CSF was added on day 2 and day 5.

### Stimulation of native P2Y_11_ in human M2 macrophages

On day 6, fully differentiated M2 macrophages were harvested, washed and seeded in 100 µl RPMI1640 supplemented with 5% FBS, 1% GlutaMAX, 10 mM HEPES, 1 mM sodium pyruvate, 1% NEAA and 1% Pen/Strep in 96-well plates (Corning/Costar, New York, USA) at a density of 5 × 10^4^ cells per well. Cells were stimulated in duplicates with the P2Y_11_ receptor agonist ATPγS (20 µM) in the presence or absence of antagonists/inhibitors and/or recombinant cytokines for 24 h. Microscopic inspection and flow cytometry-based eFluor780 exclusion was used to exclude toxicity of the inhibitors used. Supernatants were harvested and cryopreserved at − 80 °C.

### Stimulation of ectopic P2Y_11_ in human astrocytoma cells

Ectopic P2Y_11_ receptor was studied in the recombinant cell line ES-293-A (Perkin Elmer). This cell line originates from the glioma cell line 1321N1, a grade II brain astrocytoma naturally devoid of functional P2 receptors, which has been stably transfected with human P2Y_11_ receptor-coding cDNA. By using CRISPR/Cas9-mediated knockout, we previously generated a corresponding control cell line lacking P2Y_11_ receptors (P2RY11-KO) [15]. Both the P2Y_11_-expressing cell line (P2RY11) and the knockout control cell line (P2RY11-KO) were regularly sorted on a BD FACSAria at the local FACS core facility to select for presence or absence of P2Y_11_, respectively.

Upon confluence, cells were harvested, washed and 1 × 10^5^ cells were seeded in 400 µl Dulbecco’s modified Eagle’s medium (DMEM) supplemented with 10% (v/v) FBS, 1 mM sodium pyruvate, 2 mM L-alanyl-L-glutamine (Glutamax), 100 units ml^−1^ of penicillin, and 100 µg ml^−1^ of streptomycin. At this stage, FBS at 10% was required to facilitate cell attachment. P2Y_11_ stimulation (24 h) occurred in the presence of 1% FBS and in the absence of G418 (geneticin), which was otherwise used at 400 µg ml^−1^ to select for stable transfectants as well as knockouts.

### Flow cytometry

Cellular antigens were stained with fluorochrome-conjugated monoclonal (mouse) or polyclonal (rabbit, goat) antibodies. Isotype-matched control stainings were performed in parallel using the same concentration to determine unspecific background signals. First, cells were harvested, washed and stained with fixable viability dye eFluor 780 (eBioscience/Thermo Fisher Scientific) to enable dead cell discrimination. Following another washing step, cells were stained for 30 min at 4 °C in the dark in PBS (Lonza) containing 0.5% FBS and 50 µg ml^−1^ human IgG (Octapharma, Lachen, Switzerland) to block Fcγ receptors. In order to access intracellular antigens, cells were fixed with intracellular fixation buffer (Invitrogen, Waltham, MA, USA) for 30 min at room temperature in the dark after dead cell staining. Following two washing steps, cells were stained in permeabilization buffer (Nordic-MUbio, Susteren, Netherlands) for 30 min at room temperature in the dark. The following antibodies were used: rabbit polyclonal IgG antihuman P2Y_11_ receptor (bs-12071R-A-488; Bioss, Woburn, MA, USA), mouse monoclonal IgG2b antihuman CD14 (clone MϕP9, 345787-APC; BD Biosciences, Franklin Lakes, NJ, USA), mouse monoclonal IgG1 antihuman CD163 (clone GHI/61, 556018-PE; BD Biosciences), goat polyclonal IgG antihuman IL-1R1 (FAB269P-PE; R&D Systems) and mouse monoclonal IgG2b antihuman CD39 (clone TU66, 561444-FITC; BD Biosciences).

Acquisition and analysis of samples was performed on a FACSCanto II flow cytometer equipped with FACS Diva 6.1.2 software. The data were processed using FlowJo V7.2.5 software (BD Biosciences) by applying dead cell and doublet discrimination.

### Transcriptome analysis

Gene expression was examined using the NanoString’s nCounter analysis system as described previously [15]. Fully differentiated M2 macrophages were seeded in 1 ml RPMI1640 supplemented with 5% FBS, 1% GlutaMAX, 10 mM HEPES, 1 mM sodium pyruvate, 1% NEAA and 1% Pen/Strep in 12-well plates (Corning/Costar) with a density of 5 × 10^5^ cells per well. Cells were stimulated in duplicates with the P2Y_11_ receptor agonist ATPγS (20 µM) in the presence or absence of the P2Y_11_ receptor antagonist NF340 (20 µM) and/or the PDE4-selective inhibitor rolipram (10 µM). After 6 h, cells were harvested and total RNA was isolated from cell pellets using the RNeasy Plus Micro Kit (Qiagen, Hilden, Germany) according to the manufacturer’s instructions. Cell supernatants were analyzed for the presence or absence of sTNFR2 to check the successful stimulation or inhibition of the P2Y_11_ receptor. Total RNA samples were then quality checked on an Agilent Bioanalyzer 2100 (Agilent Technologies, Santa Clara, CA, USA) with an RNA 6000 Nano LabChip and samples with RNA integrity factors above 8.0 were used for further analysis. 50 ng of total RNA were used for hybridization reaction with the nCounter Host Response Panel Kit (human) according to supplier’s instructions (NanoString Technologies, Seattle, WA USA). Samples were processed at the Core Facility Molecular Biology at the Centre of Medical Research at the Medical University of Graz. The automated NanoString platform is based on fluorescent barcodes and digital readout allowing for the non-amplified measurement of 773 protein-coding mRNA sequences within one sample. The NanoString platform has been shown to be comparable with other technologies, with considerable sensitivity, reproducibility and technical robustness (https://www.nanostring.com/scientific-content/publications).

Raw data preprocessing and normalization were performed using nSolver 2.5 Software (NanoString Technologies, Seattle, WA USA) according to standard procedures (background subtraction, positive and negative controls normalization). Subsequently, gene counts were normalized to the geometric mean of the 40 reference genes. Normalized data were uploaded to Partek Genomic Suite Software v6.6 (Partek Inc, St Louis, MO). For statistical analysis, one-way ANOVA was calculated and genes with *p* < 0.05 and fold change of at least 1.5 were considered as differentially regulated.

### Cytokine measurements

Cytokine levels in cell culture supernatants were assessed by cytometric bead arrays (CBA) from BD using human CBA Flex Sets for VEGF and sTNFR2 according to the manufacturer’s instructions. Supernatants were analyzed on a FACSCanto II flow cytometer and FCAP Array 1.0.1 software (BD).

The levels of CCL20 (MIP-3α) in cell culture supernatants were determined by ELISA using the Human MIP-3 alpha ELISA Kit (RayBiotech, Peachtree Corners, GA, USA) or the Human CCL20/MIP-3 alpha DuoSet (R&D Systems) according to the manufacturer’s instructions. Measurements were carried out on an Elx800 universal microplate reader (BioTek Instruments/Agilent, Winooski, VT, USA) and results were processed using the Gen5 3.09 data analysis software (BioTek Instruments/Agilent).

### Data and statistical analysis

The data are presented as mean values ± SD. Sample sizes and experimental replicates are indicated in figure legends. Statistical analyses were conducted with the GraphPad Prism software (version 9). Statistical significance was determined by ordinary one-way ANOVA including Bonferroni correction. An output of *p* < 0.05 was accepted as significantly different in all tests. Significance levels are: *p < 0.05; **p < 0.01; ***p < 0.001; ****p < 0.0001.

## Results

### Transcriptome profiling of native P2Y_11_ in human M2 macrophages reveals a strong signature of an anti-inflammatory and proangiogenic P2Y_11_/IL-1R crosstalk

**Fig. 1:**
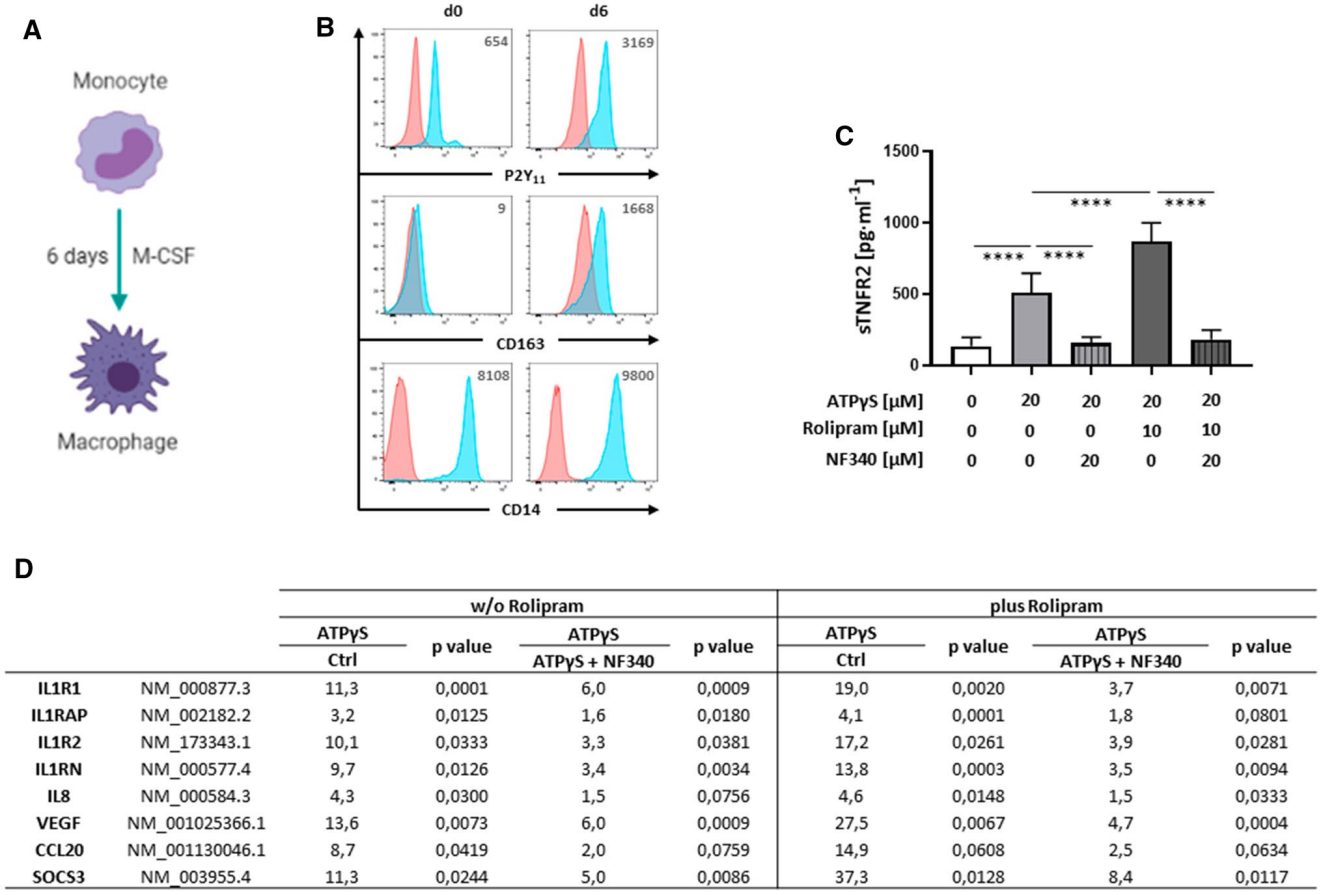
P2Y_11_ activation on human M2 macrophages elicits an anti-inflammatory and pro-angiogenic response including the upregulation of genes related to IL-1 signaling. Primary human monocytes were obtained from peripheral blood mononuclear cells (PBMCs) by magnetic-activated cell sorting (MACS) using CD14 microbeads. **A** M2 macrophages were differentiated by culturing isolated monocytes in the presence of M-CSF (50 ng·ml^−1^) for 6 days. **B** The phenotype of M2 polarized macrophages was determined by flow cytometry with CD163 serving as an M2 marker antigen. Numbers represent mean fluorescence intensities (MFIs) of the respective staining after subtraction of isotype control MFIs. **C** M2 macrophages were treated for 24 h with the P2Y_11_ receptor agonist ATPγS (20 µM) in the presence or absence of the PDE4 inhibitor rolipram (10 µM). sTNFR2 levels were measured in cell culture supernatants. The selective P2Y_11_ antagonist NF340 (20 µM) was used to confirm that agonist-mediated responses were specific to P2Y_11_ receptor stimulation (*n* = 5). ****p < 0.0001. **D** Gene activation in response to P2Y_11_ receptor stimulation was studied using NanoString analysis. The data are expressed as fold change over controls. M2 macrophages were cultured for 6 h in the presence of the P2Y_11_ receptor agonist ATPγS (20 µM) either alone or in combination with the PDE4 inhibitor rolipram (10 µM). NF340 (20 µM) was used to confirm that agonist-mediated transcriptional changes were specific to P2Y_11_ receptor stimulation. Untreated cells served as a negative control. *p* values shown were calculated by one-way ANOVA

To examine P2Y_11_ downstream signaling events, we set out to establish the transcriptional profile of P2Y_11_ activation. Although our previous study focused on ectopic P2Y_11_ in a recombinant cell system [15], we here investigated native P2Y_11_ in its natural environment. Human monocytes upregulate P2Y_11_ along with the M2 marker CD163 during macrophage differentiation induced by M-CSF (Fig. 1A, B) [8] and release sTNFR2 in response to triggering of P2Y_11_ with its specific agonist ATPγS (20 µM) (Fig. 1C) [15]. NF340 (20 µM), which is currently the most useful antagonist at the P2Y_11_ receptor [10], effectively prevented P2Y_11_-induced and rolipram-enhanced sTNFR2 release. Rolipram inhibits PDE4 and thus increases the levels of intracellular cAMP [22]. Using this well-defined experimental cell system [15], we analyzed gene expression with NanoString technology and found a distinct pattern of P2Y_11_/cAMP-driven reprogramming in human M2 macrophages (Fig. 1D). Activation of native P2Y_11_ caused upregulation of the IL-1R family members IL1R1, IL1RAP and IL1R2 [23] in a manner very similar to that previously observed with ectopic P2Y_11_ [15]. IL1RN, the gene encoding IL-1R antagonist, was also activated. The activating IL-1R is a complex of two chains, the IL-1α/IL-1ß-binding chain IL-1R1 (encoded by IL1R1) and the accessory protein IL-1R3 (encoded by IL1RAP), which is responsible for signaling [23].

NLRP3 inflammasome activation in macrophages requires two signals [24]. The first signal, also known as priming signal, can be provided by microbial or endogenous molecules that trigger toll-like receptors. The second signal, causing inflammasome activation, can be delivered by ATP-activated P2X_7_. Intriguingly, both priming (toll-like receptors) and secondary signals (P2RX7) were downregulated upon P2Y_11_ activation along with NLRP3, CASP1 and PYCARD, which encode the NLRP3 inflammasome components NLRP3, pro-caspase-1 and the adapter protein PYCARD (ASC) (Table 1). Moreover, in support of an efficient activation of P2Y_11_, ENTPD1, the gene encoding the ecto-ATPase CD39, was downregulated.

**Table 1:** P2Y_11_-induced and rolipram-enhanced downregulation of ENTPD1 (CD39) and pro-inflammatory genes in human M2 macrophages

		w/o Rolipram				plus Rolipram			
		**ATPγS**	***p* value**	**ATPγS + NF340**	***p* value**	**ATPγS**	**p value**	**ATPγS + NF340**	***p* value**
		**Ctrl**		**ATPγS**		**Ctrl**		**ATPγS**	
ENTPD1	NM_001098175.1	2.0	0.030	1.6	0.0187	2.2	0.0225	1.4	0.0168
P2RX7	NM_002562.5	7.1	0.004	4.2	0.0013	29.7	0.0144	13.1	0.0153
NLRP3	NM_001079821.2	2.1	0.082	2.2	0.0261	2.1	0.0349	1.8	0.0405
CASP1	NM_033294.3	2.3	0.017	2.2	0.0190	4.6	0.0014	3.6	0.0030
PYCARD	NM_013258.3	2.8	0.051	2.2	0.1065	4.2	0.0262	2.9	0.0074
TLR5	NM_003268.5	2,9	0.031	2.2	0.0028	7.2	0.0154	4.7	0.0068
TLR7	NM_016562.3	18.0	0.015	8.1	0.0008	42.2	0.0171	11.1	0.0202
TLR8	NM_016610.2	2.4	0.008	1.6	0.0135	3.9	0.0004	2.5	0.0174

Human M2 macrophages were stimulated with the P2Y_11_ agonist ATPγS (20 µM) in the presence or absence of the PDE4 inhibitor rolipram (10 µM). The P2Y_11_ antagonist NF340 (20 µM) was used to confirm that ATPγS-induced responses were specific to P2Y_11_ receptor stimulation. mRNA expression was quantified using NanoString technology. Data are expressed as fold change over controls

The P2Y_11_ transcriptome also contained the signature of an angiogenic response. In addition to the pro-angiogenic chemokine CXCL8 (IL8), which previously emerged from the transcriptional profile of ectopic P2Y_11_ [15], VEGFA and CCL20, encoding vascular endothelial growth factor and chemokine (C−C motif) ligand 20, respectively, were also found to be P2Y_11_ target genes in M2 macrophages (Fig. 1D). The observed P2Y_11_-driven anti-inflammatory and pro-angiogenic transcriptional responses were even more pronounced, when PDE4-mediated cAMP degradation was inhibited with rolipram. Finally, SOCS3, which encodes suppressor of cytokine signaling 3, was also upregulated by P2Y_11_ signaling (Fig. 1D). This was of particular interest, because SOCS3 is a known feedback regulator of IL-1 signaling [25] and an anti-inflammatory target gene of the cAMP-binding effector Epac1 [18, 19].

NanoString-based gene expression analysis reveals mRNA copy numbers for each individual gene and by using a set of negative control probes, the general gene expression threshold can be determined. We thus found that IL1R2, CCL20 and SOCS3 genes were silent in human M2 macrophages but were activated upon P2Y_11_ activation (not shown).

**Fig. 2:**
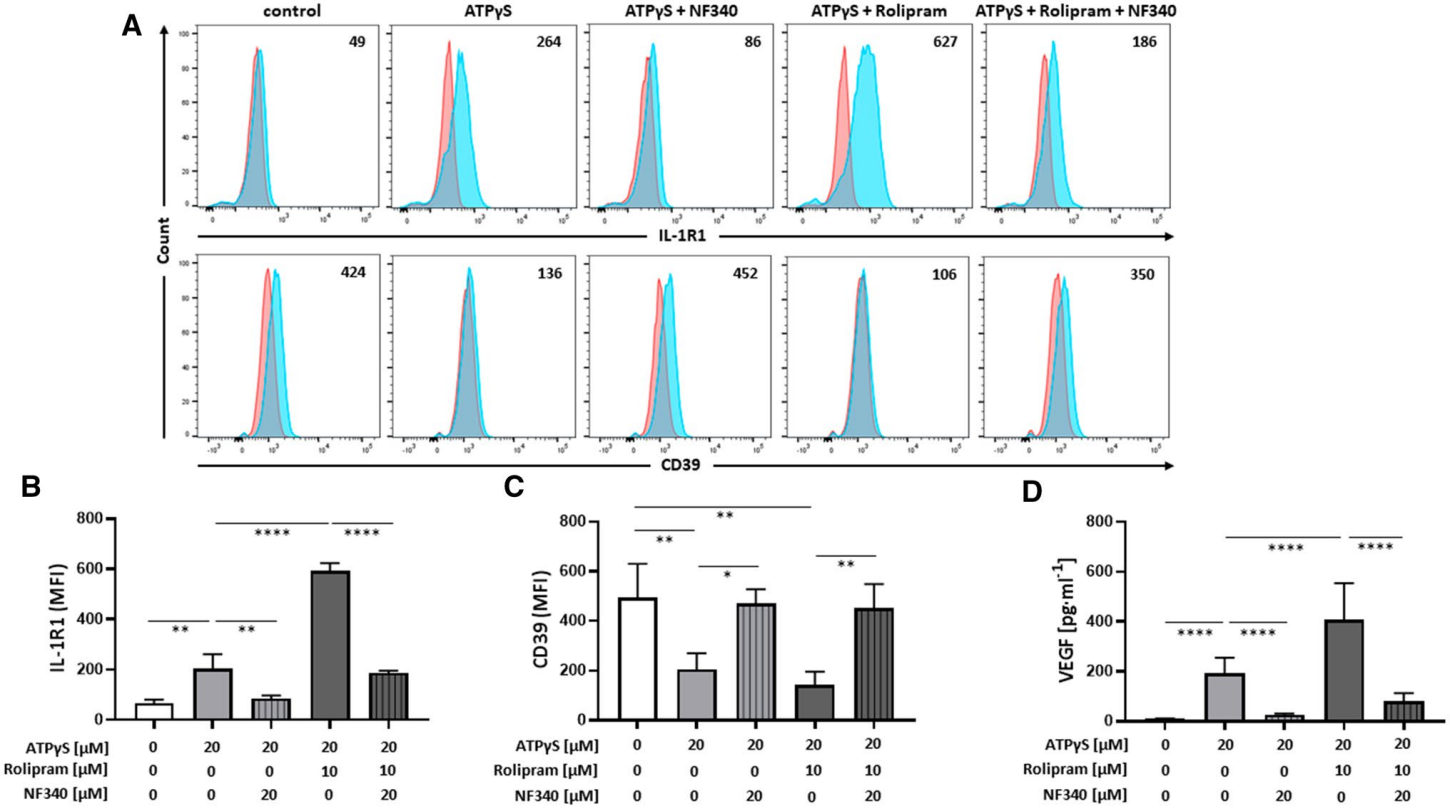
P2Y_11_ activation on human M2 macrophages causes IL-1R upregulation and CD39 downregulation as well as VEGF secretion. **A–C** The regulation of IL-1R1 and the ecto-ATPase CD39 at the protein level was examined by flow cytometry. M2 macrophages were treated for 24 h with the P2Y_11_ receptor agonist ATPγS (20 µM) in the presence or absence of the PDE4 inhibitor rolipram (10 µM). The antagonist NF340 (20 µM) was used to confirm that agonist-mediated responses were specific to P2Y_11_ receptor stimulation. **A** Representative FACS histograms of IL-1R1 and CD39 expression from differentially treated M2 macrophages. Numbers represent mean fluorescence intensities (MFIs) of the respective staining after subtraction of isotype control MFIs. **B, C** Quantification of IL-1R1 (**B**) and CD39 (**C**) expression levels from differentially treated M2 macrophages (*n* = 3). *p < 0.05, **p < 0.01, ****p < 0.0001. **D** M2 macrophages were treated for 24 h with the P2Y_11_ receptor agonist ATPγS (20 µM) in the presence or absence of the PDE4 inhibitor rolipram (10 µM). VEGF levels were measured in cell culture supernatants. NF340 (20 µM) was used to confirm that agonist-mediated responses were specific to P2Y_11_ receptor stimulation (*n* = 5). ****p < 0.0001

To validate selected candidates emerging from the NanoString analysis, we performed flow cytometric analyses and cytokine bead arrays. Upregulation of IL-1R1 expression on the surface of human M2 macrophages in response to P2Y_11_ activation with ATPγS is shown in Fig. 2A, B. P2Y_11_ stimulation in the presence of rolipram caused a significant further increase of IL-1R1 expression. NF340 effectively prevented both, P2Y_11_-driven and rolipram-boosted upregulation of IL-1R1 (Fig. 2A, B). Conversely, ENTPD1-encoded CD39, which acts as an ecto-ATPase on the surface of macrophages and other cell types to degrade extracellular ATP [26], was downregulated during P2Y_11_ signaling (Fig. 2A/C), thus facilitating the prolonged activation of P2Y_11_ by its physiological agonist ATP. VEGF secretion could also be validated (Fig. 2D) and showed a pattern almost identical to that of sTNFR2 release (Fig. 1C).

### ATPγS-induced secretory responses are specific to P2Y_11_ and additionally depend on Ca^2+^ and protein kinase C

ATPγS is a selective P2Y_11_ receptor agonist [7]. According to the IUPHAR/BPS Guide to Pharmacology [27], ATPγS is primarily considered a full agonist at the P2Y_11_ receptor. In some recombinant cell systems, it may however also act as a partial agonist at P2Y_1_ [28] and as a full agonist at P2Y_13_ [29]. We therefore performed additional experiments using well-established antagonists of P2Y_1_ (MRS 2500) [30] and P2Y_13_ (MRS 2211) [31]. Importantly, both had little effect on ATPγS-induced and rolipram-enhanced VEGF secretion and sTNFR2 release (Fig. S1). In contrast, NF340 effectively prevented these responses. Therefore, we considered ATPγS a bona fide, potent, and selective P2Y_11_ agonist in human M2 macrophages.

**Fig. 3:**
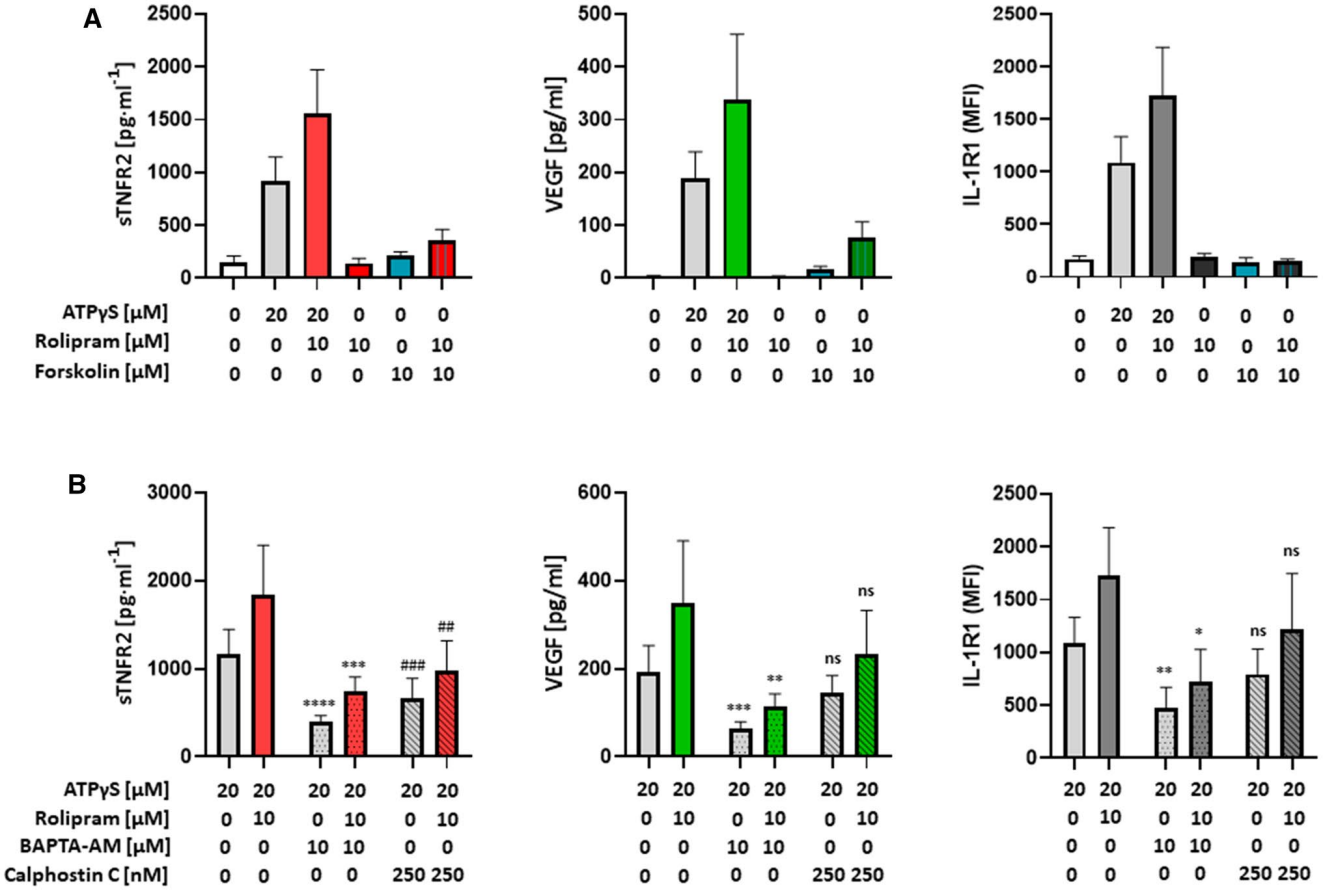
P2Y_11_-mediated sTNFR2 release and VEGF secretion as well as IL-1R upregulation depend on Ca^2+^ and  protein kinase C, but are differentially regulated by cAMP elevation. **A** M2 macrophages were treated for 24 h with the P2Y_11_ receptor agonist ATPγS (20 µM) in the presence or absence of the PDE4 inhibitor rolipram (10 µM). In addition, cells were treated either with the PDE4 inhibitor rolipram (10 µM), the direct AC activator forskolin (10 µM) or with the combination of both. sTNFR2 and VEGF were measured in cell culture supernatants and IL-1R1 MFIs were determined by flow cytometry. Mean IL-1R1 MFI values are shown (*n* = 3). **B** M2 macrophages were treated for 24 h with the P2Y_11_ receptor agonist ATPγS (20 µM) in the presence or absence of the PDE4 inhibitor rolipram (10 µM), either with or without the Ca^2+^ chelator BAPTA-AM (10 µM) or the protein kinase C inhibitor calphostin C (250 nM). sTNFR2 and VEGF were measured in cell culture supernatants and IL-1R1 MFIs were determined by flow cytometry. Mean IL-1R1 MFI values are shown (*n* = 3). *p < 0.05, **p < 0.01, ***p < 0.001, ****p < 0.0001, ^##^*p* < 0.01, ^###^*p* < 0.001

The observed stimulatory effects of the PDE4 inhibitor rolipram clearly implicated cAMP signaling in sTNFR2 release, VEGF secretion and IL-1R upregulation but also raised the question of whether cAMP elevation is sufficient to induce these responses. To address this point, we directly activated AC with forsoklin. Forskolin had a very modest stimulatory effect on VEGF secretion and sTNFR2 release, which was enhanced in the presence of rolipram (Fig. 3A). However, forskolin was much less effective compared to ATPγS, suggesting that ATPγS-activated P2Y_11_ couples to additional signaling pathways. Rolipram alone had no effect. In contrast to VEGF secretion and sTNFR2 release, forskolin failed to upregulate IL-1R even in the presence of rolipram (Fig. 3A), indicating that cAMP elevation is not sufficient to increase IL-1R expression.

SQ22536 is probably the most widely used AC inhibitor in intact cell studies [32]. However, its potency is rather low. We tested SQ22536 in concentrations of up to 100 µM but did not observe significant inhibition of sTNFR2 release and VEGF secretion. We did not test higher concentrations as they might cause off-target effects [32].

The differential coupling of P2Y_11_ to G proteins not only leads to the elevation of intracellular cAMP but may also cause activation of Ca^2+^ and protein kinase C (PKC) signaling [16]. To investigate these second messengers in P2Y_11_-driven responses, we used the Ca^2+^ chelator BAPTA-AM [33] and the PKC inhibitor calphostin C [34]. BAPTA-AM significantly inhibited ATPγS-induced and rolipram-enhanced sTNFR2 release and VEGF secretion (Fig. 3B). Calphostin C also inhibited both, sTNFR2 release and VEGF secretion, but only sTNFR2 inhibition was significant.

In addition to cAMP-elevating agents such as rolipram (Figs. 1 and 2) or prostaglandin E2 [35], IL-1R expression is known to be stimulated by IL-1, Ca^2+^ and PKC [35–37]. Accordingly, BAPTA-AM significantly suppressed ATPγS-induced and rolipram-enhanced IL-1R upregulation (Fig. 3B). Calphostin C also showed an inhibitory effect, which was however less potent and not significant. Collectively, our data clearly implicate cAMP and Ca^2+^ in ATPγS-induced and rolipram-enhanced P2Y_11_ signaling in human M2 macrophages.

### P2Y_11_-induced IL-1R upregulation translates into increased IL-1 responsiveness of human M2 macrophages: PDE4 inhibition potentiates the secretory response

**Fig. 4:**
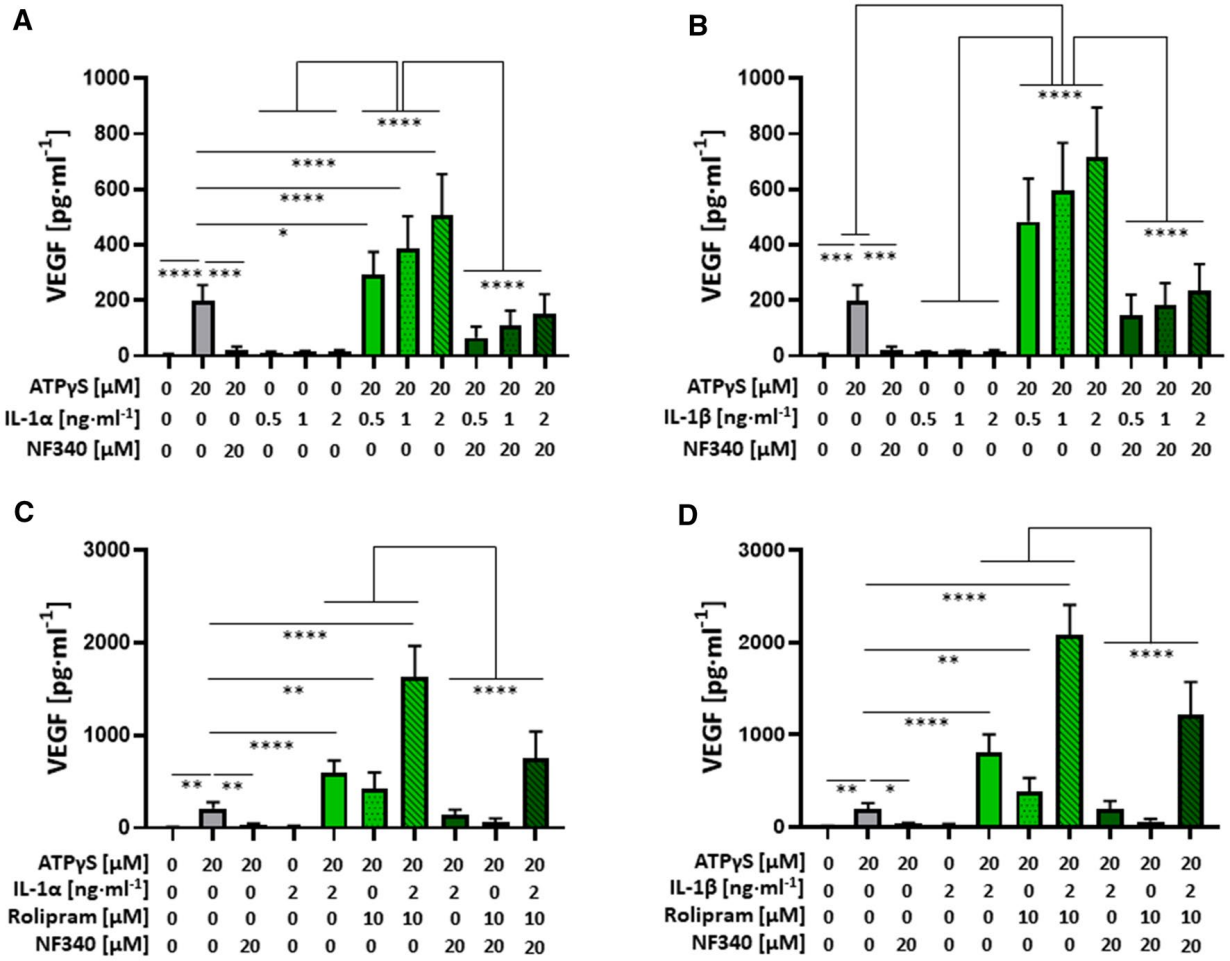
P2Y_11_ activation on human M2 macrophages increases IL-1 responsiveness and enhances VEGF release, which is even more pronounced when intracellular cAMP levels are raised through PDE inhibition. **A–D** M2 macrophages were treated for 24 h with increasing doses of IL-1α (**A, C**) or IL-1β (**B, D**), either alone or in combination with the P2Y_11_ receptor agonist ATPγS (20 µM), in the presence or absence of the PDE4-selective inhibitor rolipram (10 µM) (**C, D**). VEGF levels were measured in cell culture supernatants. NF340 (20 µM) was used to confirm that agonist-mediated responses were specific to P2Y_11_ receptor stimulation (*n* = 3). *p < 0.05, **p < 0.01, ***p < 0.001, ****p < 0.0001

Next, we investigated the regulation of P2Y_11_-induced VEGF secretion by exogenous IL-1 and by intracellular cAMP. The release of sTNFR2 served as a control in these experiments. IL-1α and IL-1ß moderately enhanced sTNFR2 release from M2 macrophages in a dose-dependent manner (Fig. S2). However, the induction of VEGF secretion by IL-1 cytokines was ineffective (Fig. 4). In contrast, the concurrent stimulation of P2Y_11_ and IL-1R resulted in the synergistic upregulation of both, sTNFR2 release and VEGF secretion (Fig. S2 and Fig. 4). The synergistic enhancement of the IL-1R—driven secretory response by ATPγS could effectively be inhibited with the P2Y_11_ antagonist NF340. Inducing the accumulation of intracellular cAMP by PDE4 blockade with rolipram potentiated the P2Y_11_/IL-1R—driven secretion of sTNFR2 and VEGF (Fig. S2 and Fig. 4C, D). The synergy of the P2Y_11_/IL-1R crosstalk was more pronounced for VEGF secretion than for sTNFR2 release.

The late stage accumulation of sTNFR2 and VEGF at 24 h might be considered part of an anti-inflammatory process that occurs in response to prolonged IL-1R activation and may therefore represent secondary, negative feedback signaling to control the initial pro-inflammatory IL-1R response. To address this point, we have performed kinetic analyses of sTNFR2 release and VEGF secretion (6, 12, and 24 h). P2Y_11_ signaling induced by ATPγS alone stimulated sTNFR2 release and VEGF secretion, which were clearly detectable at 6 h and continued to increase at 12 h but no longer at 24 h (Fig. S3). Although rolipram had little or no effect on ATPγS-induced secretory responses at 6 h, it modestly increased sTNR2 levels at 12 h and 24 h, and more effectively, the levels of VEGF at 24 h. IL-1 co-stimulation had relatively little effect at 6 h, but strongly enhanced the ATPγS-driven release of sTNFR2 as well as the secretion of VEGF, both at 12 h and at 24 h. Combined P2Y_11_ co-stimulation with rolipram and IL-1 resulted in the highest levels of sTNFR2 and VEGF, however, 24 h were required to express the full co-stimulatory effect.

Taken together, these observations suggested that the observed sTNFR2 release and VEGF secretion is the result of an immediate, direct response and not secondary to strong pro-inflammatory IL-1R signaling. Early P2Y_11_ canonical signaling causes IL-1R upregulation (Figs. 1D and 2A) and thus facilitates IL-1 co-stimulation, which, however, depends on protracted signaling and possibly also on enhanced gene expression [38].

VEGF has been implicated in the stimulation of ADAM17-dependent shedding of cell surface receptors [39], raising the possibility that P2Y_11_-induced VEGF promotes ADAM17-dependent sTNFR2 release. To test this, we used bevacizumab, a humanized anti-VEGF monoclonal antibody. Bevacizumab effectively neutralized P2Y_11_-induced VEGF, however, this had no effect on P2Y_11_-induced sTNFR2 release (Fig. S4), excluding the possibility that VEGF stimulates ADAM17 in our setting.

### The cAMP binding effector protein Epac1 controls P2Y_11_-induced sTNFR2 release

**Fig. 5:**
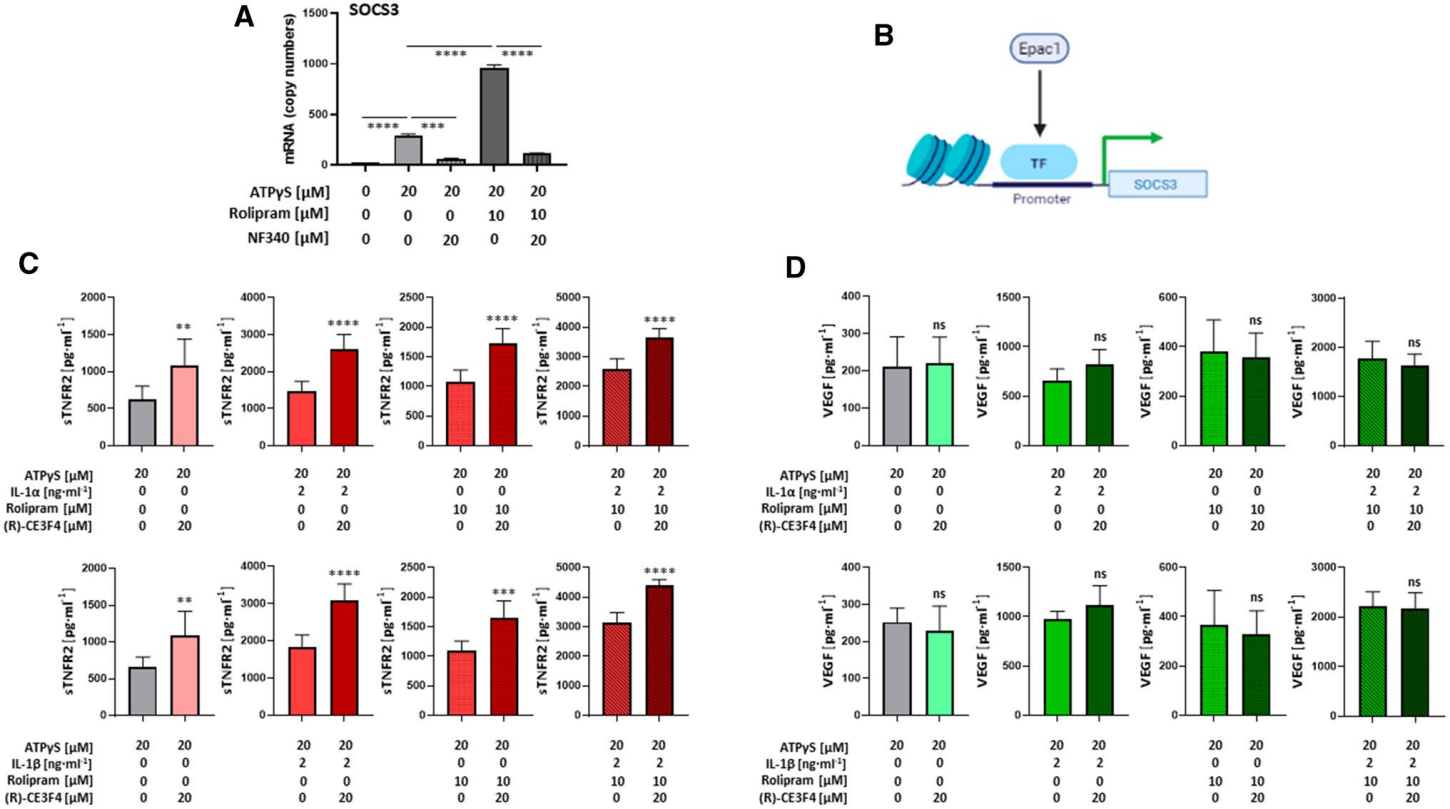
The P2Y_11_ receptor-mediated release of sTNFR2, but not of VEGF, is controlled by the cAMP effector binding protein Epac1. **A** P2Y_11_ receptor signaling upregulates the expression of suppressor of cytokine signaling 3 (SOCS3). M2 macrophages were cultured for 6 h in the presence of the P2Y_11_ receptor agonist ATPγS (20 µM) either alone or in combination with the PDE4 inhibitor rolipram (10 µM), and the copy numbers of SOCS3 mRNA were determined using NanoString technology. NF340 (20 µM) was used to confirm that agonist-mediated changes were specific to P2Y_11_ receptor stimulation. ***p < 0.001, ****p < 0.0001. **B** Graphical illustration showing that SOCS3 is a target gene of Epac1. **C**, **D** M2 macrophages were treated for 24 h with the P2Y_11_ receptor agonist ATPγS (20 µM) either alone or in combination with IL-1α (2 ng·ml^−1^) or IL-1β (2 ng·ml^−1^) in the presence or absence of the PDE4-selective inhibitor rolipram (10 µM). In addition, all treatments were carried out in the presence or absence of the selective Epac1 inhibitor (R)-CE3F4 (20 µM). sTNFR2 (**C**) and VEGF (**D**) levels were measured in cell culture supernatants (n = 3). **p < 0.01, ***p < 0.001, ****p < 0.0001

The strong P2Y_11_-induced and rolipram-enhanced upregulation of SOCS3 (Fig. 5A), which is a target of Epac1 (Fig. 5B) [18], suggested an involvement of the cAMP-binding effector protein Epac1. To test this possibility, we used the (R)-enantiomer of CE3F4, which is a preferential inhibitor of Epac1 [40]. Epac1 inhibition with (R)-CE3F4 enhanced the P2Y_11_-mediated release of sTNFR2 (Fig. 5C) but had no effect on VEGF secretion (Fig. 5D). The release of sTNFR2 induced by ATPγS alone or ATPγS plus either IL-1 or rolipram was significantly enhanced by Epac1 inhibitor (R)-CE3F4. In contrast, the strong sTNFR2 release triggered by ATPγS plus IL-1 and rolipram appeared to approach saturation as (R)-CE3F4 was much less effective in enhancing it (Fig. 5C).

**Fig. 6:**
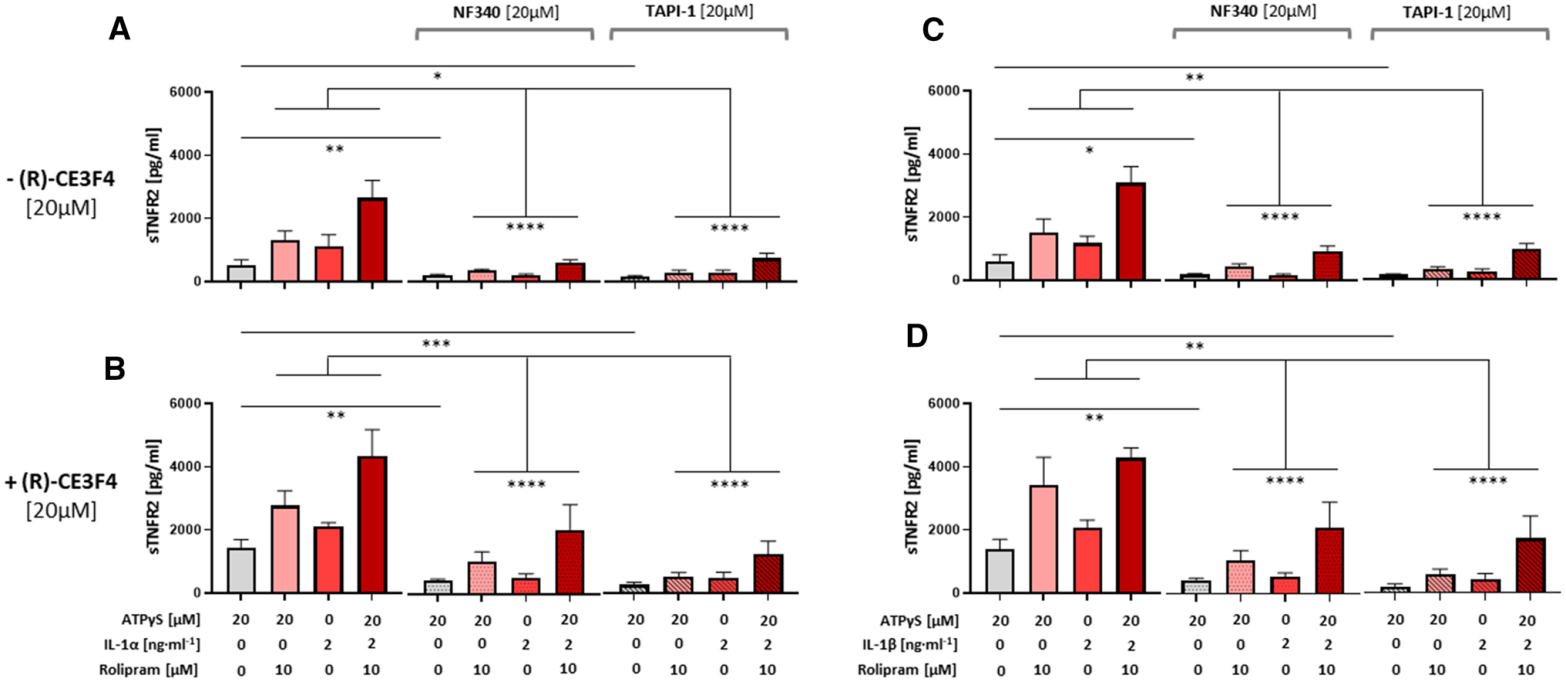
The Epac1 inhibitor-mediated enhancement of the P2Y_11_-driven release of sTNFR2 depends on TACE/ADAM17. **A–D** M2 macrophages were treated for 24 h with the P2Y_11_ receptor agonist ATPγS (20 µM) either alone (**A**, **C**) or in combination with the selective Epac1 inhibitor (R)-CE3F4 (20 µM) (**B**, **D**), in the presence or absence of IL-1α (2 ng·ml^−1^) (**A**, **B**) or IL-1β (2 ng·ml^−1^) (**C**, **D**), with or without the PDE4-selective inhibitor rolipram (10 µM) (**A–D**). TAPI-1 (20 µM) was used to verify an involvement of TACE/ADAM17 in the shedding of sTNFR2. sTNFR2 levels were determined in cell culture supernatants using CBA. NF340 (20 µM) was used to confirm that agonist-mediated responses were specific to P2Y_11_ receptor stimulation (*n* = 3). *p < 0.05, **p < 0.01, ***p < 0.001, ****p < 0.0001

Next, we examined the role of ADAM17 in the P2Y_11_-driven and (R)-CE3F4-enhanced secretory response. Both, P2Y_11_ inhibitor NF340 (20 µM) and ADAM17 inhibitor TAPI-1 (20 µM) suppressed the ATPγS/IL-1-induced and (R)-CE3F4-enhanced release of sTNFR2 from M2 macrophages (Fig. 6). However, although NF340 also prevented the ATPγS/IL-1-induced secretion of VEGF, TAPI-1 as expected had no effect on this response (Fig. S5).

### P2Y_11_/IL-1R crosstalk activates CCL20: PDE4 inhibition potentiates and Epac1 regulates the secretory response

**Fig. 7:**
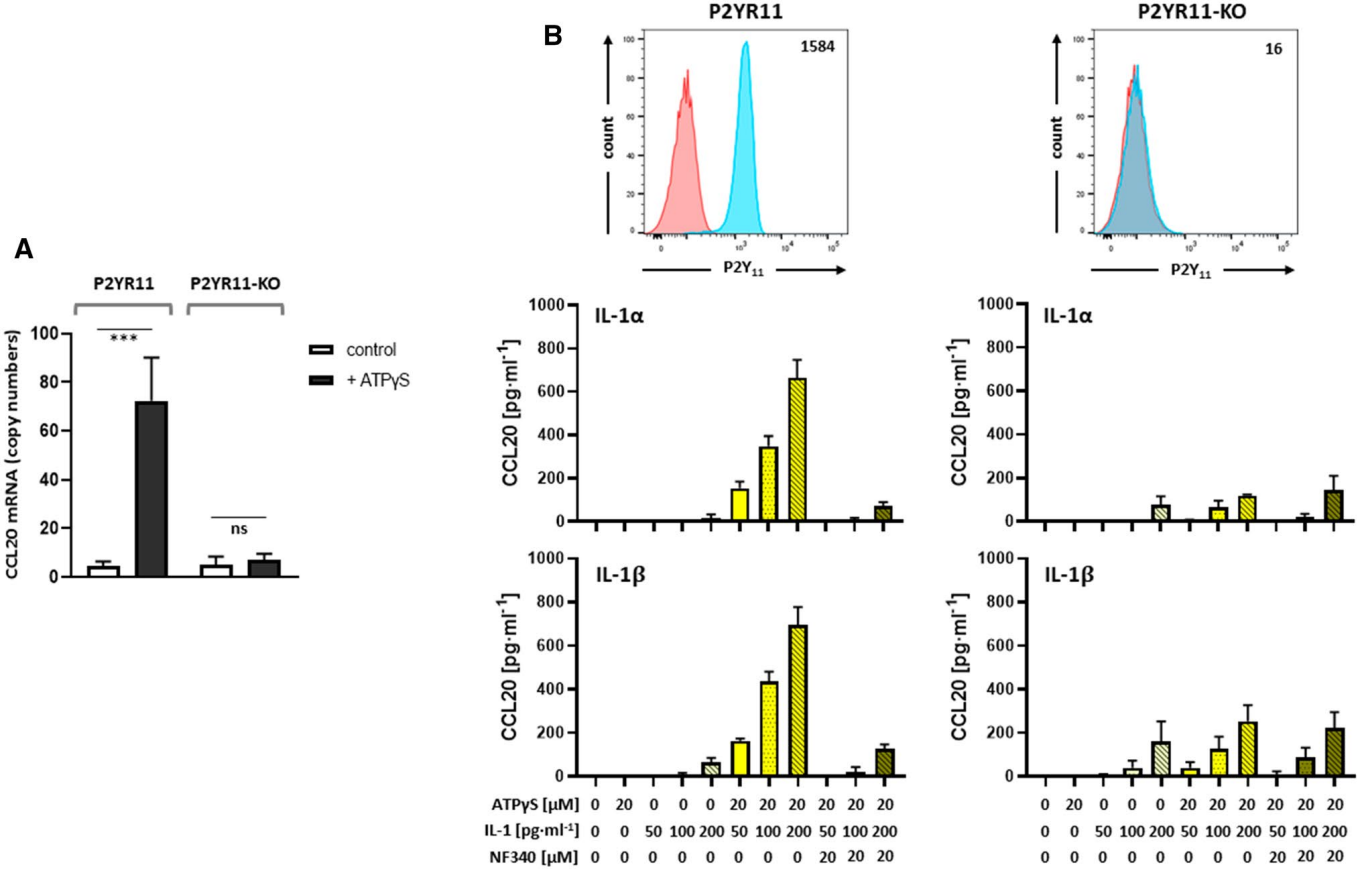
Ectopic P2Y_11_ and IL-1R synergize to induce CCL20 secretion. **A** Transcriptional activation of CCL20 in response to ectopic P2Y_11_ activation was examined using NanoString technology. P2RY11-transfected (P2RY11) or P2RY11-knockout (P2RY11-KO) astrocytoma cells were cultured for 6 h in the presence of the P2Y_11_ agonist ATPγS at 20 µM. **B** Surface expression of P2Y_11_ receptors was measured by flow cytometry in the recombinant cell line (P2YR11; left panel) and in the corresponding knockout control (P2YR11-KO; right panel). Numbers represent mean fluorescence intensities (MFIs) of P2Y_11_ staining after subtraction of isotype control MFIs. P2RY11-transfected cells and P2RY11 knockout cells (P2RY11-KO) were treated for 24 h with increasing doses of IL-1α or IL-1β, either alone or in combination with the P2Y_11_ receptor agonist ATPγS (20 µM). CCL20 levels were measured in cell culture supernatants. NF340 (20 µM) was used to confirm that agonist-mediated transcriptional changes were specific to P2Y_11_ receptor stimulation (*n* = 3). ***p < 0.001

We have also performed mRNA profiling of recombinant P2Y_11_ in a side-by-side stimulation of astrocytoma cells with either P2RY11 overexpression (P2RY11) or P2RY11 knockdown (P2RY11-KO) [15]. ATPγS induced strong upregulation of CCL20 mRNA in P2RY11 cells but not in P2RY11-KO cells (Fig. 7A). In fact, CCL20 was among the most highly activated genes in response to ectopic P2Y_11_ stimulation in astrocytoma cells. However, despite the much stronger signature of IL-1 signaling in P2RY11 cells [15], we found that ATPγS alone failed to stimulate CCL20 production and that exogenous IL-1α or IL-1ß were still required (Fig. 7B). Both, P2RY11 and P2RY11-KO cells produced low levels of CCL20 in response to increasing doses of IL-1. However, the synergistic upregulation with ATPγS was only observed in P2RY11 cells. Moreover, ATPγS/IL-1-driven CCL20 production in P2RY11 cells was effectively suppressed by NF340.

**Fig. 8:**
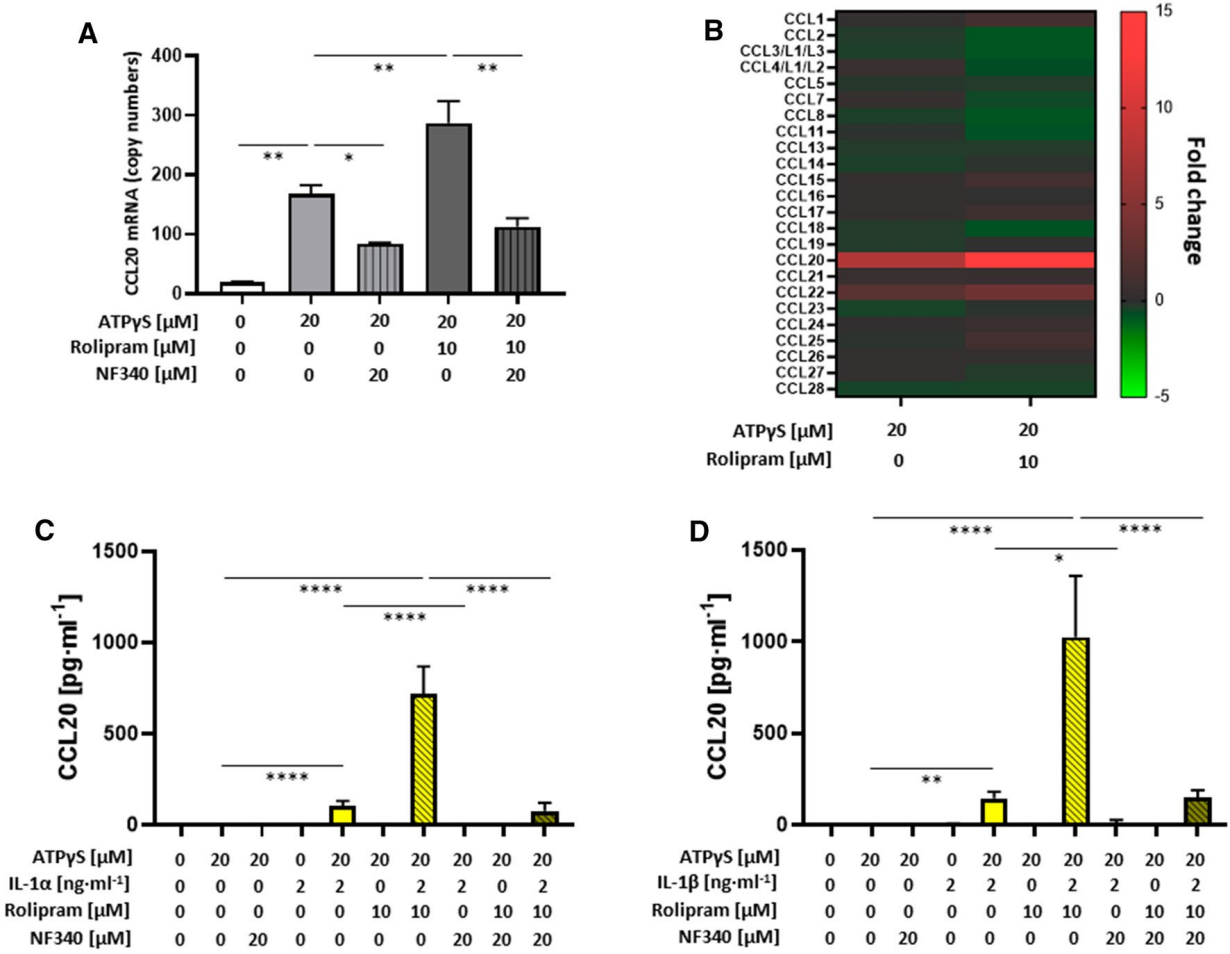
Native P2Y_11_ and IL-1R synergize to selectively induce CCL20 secretion. **A**, **B** Transcriptional activation of CCL20 in response to native P2Y_11_ activation was examined using NanoString technology. M2 macrophages were cultured for 6 h in the presence of the P2Y_11_ receptor agonist ATPγS (20 µM) either alone or in combination with the PDE4 inhibitor rolipram (10 µM). NF340 (20 µM) was used to confirm that agonist-mediated changes were specific to P2Y_11_ receptor stimulation. **A** The copy numbers of CCL20 mRNA were determined using NanoString technology. For statistical analysis, One-Way ANOVA was calculated. **B** Heat map displaying the fold-change of CCL chemokine mRNA expression in human M2 macrophages in response to P2Y_11_ receptor stimulation with ATPγS (20 µM) either alone or in combination with the PDE4 inhibitor rolipram (10 µM). **C**, **D** M2 macrophages were treated for 24 h with the P2Y_11_ receptor agonist ATPγS (20 µM) either alone or in combination with IL-1α (2 ng·ml^−1^) (**C**) or IL-1β (2 ng·ml^−1^) (**D**), in the presence or absence of the PDE4-selective inhibitor rolipram (10 µM). CCL20 levels were measured in cell culture supernatants. NF340 (20 µM) was used to confirm that agonist-mediated responses were specific to P2Y_11_ receptor stimulation (*n* = 3). *p < 0.05, **p < 0.01, ****p < 0.0001

In human M2 macrophages, ATPγS also induced strong upregulation of CCL20 mRNA and rolipram enhanced this response (Fig. 8A). Both, ATPγS-induced and rolipram-enhanced CCL20 gene activation could be inhibited with NF340. It is important to note that only CCL20 was upregulated among the 24 CCL chemokines analyzed (Fig. 8B), implying a strong selectivity in P2Y_11_-induced CCL chemokine activation. The synergy between P2Y_11_ and IL-1R was extreme for CCL20 secretion. ATPγS or IL-1 alone failed to induce CCL20 secretion (Fig. 8C, D). The combination of ATPγS and IL-1 was required to induce low-level secretion of CCL20. Raising cAMP through PDE4 suppression appeared to be the critical step as it caused robust upregulation of P2Y_11_/IL-1R—driven CCL20 secretion (Fig. 8C, D).

**Fig. 9:**
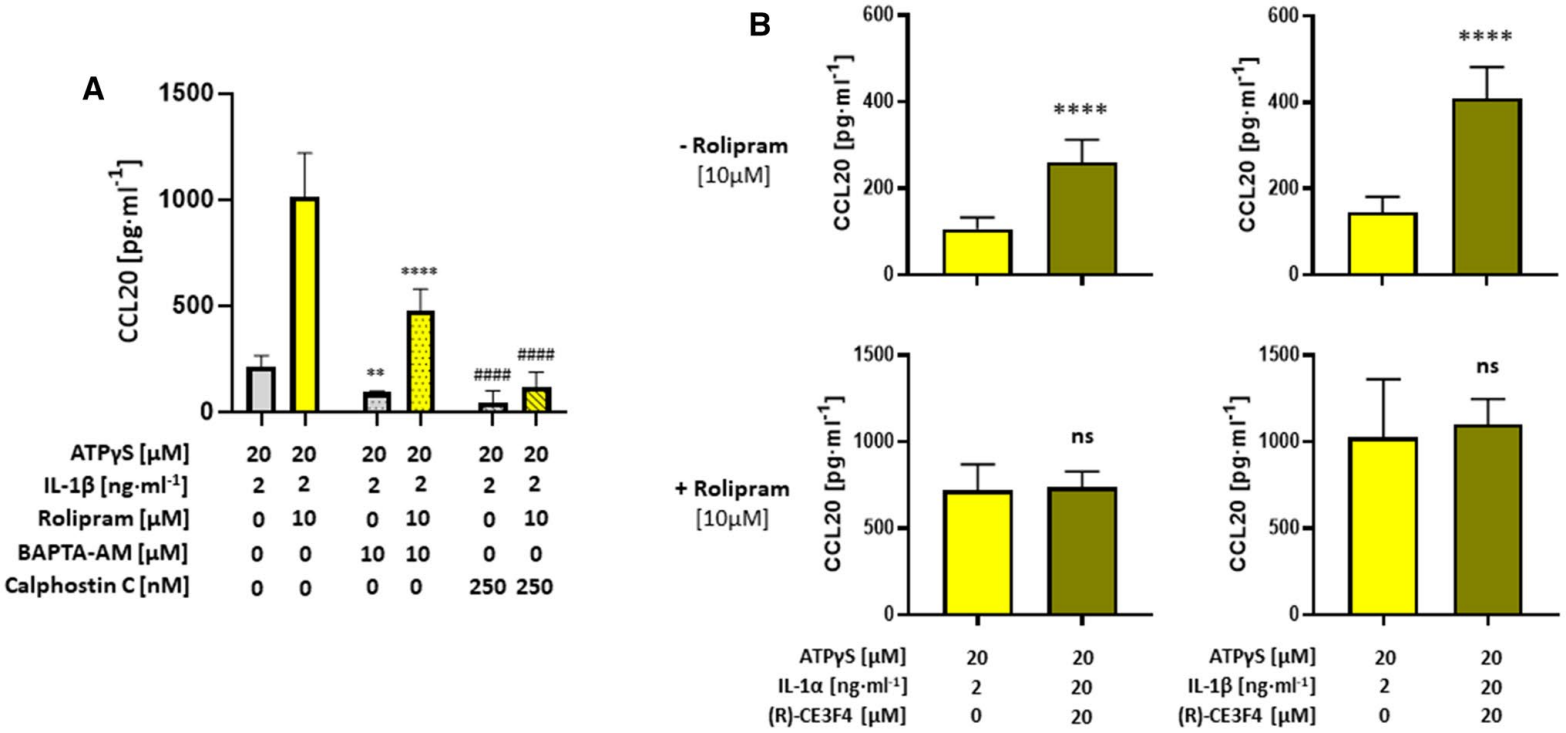
The P2Y_11_/IL-1R-mediated secretion of CCL20 strongly depends on PKC signaling as well as on cAMP and Ca^2+^, while Epac1 inhibition enhances the P2Y_11_/IL-1R-mediated secretion of CCL20, an effect which is abolished when intracellular cAMP levels are raised by PDE4 inhibition.** A** M2 macrophages were treated for 24 h with the P2Y_11_ receptor agonist ATPγS (20 µM) either alone or in combination with IL-1β (2 ng·ml^−1^), in the presence or absence of the PDE4-selective inhibitor rolipram (10 µM). In addition, all treatments were conducted with or without the Ca^2+^ chelator BAPTA-AM (10 µM) or the protein kinase C inhibitor calphostin C (250 nM). CCL20 levels were measured in cell culture supernatants (*n* = 3). **B** M2 macrophages were treated for 24 h with the P2Y_11_ receptor agonist ATPγS (20 µM) either alone or in combination with IL-1α (2 ng·ml^−1^) or IL-1β (2 ng·ml^−1^), in the presence or absence of the PDE4-selective inhibitor rolipram (10 µM). In addition, all treatments were performed with or without the selective Epac1 inhibitor (R)-CE3F4 (20 µM). CCL20 levels were determined in cell culture supernatants (*n* = 3). **p < 0.01, ****p < 0.0001, ^####^*p* < 0.0001

Similar to VEGF secretion and sTNFR2 release, CCL20 production not only depended on cAMP but also on Ca^2+^ and PKC signaling (Fig. 9A). While BAPTA-AM partially inhibited CCL20 production driven by ATPγS/IL-1 and ATPγS/IL-1/rolipram, Calphostin C abrogated the response almost completely, confirming the particularly strong PKC dependence of CCL20 biosynthesis [41] and suggesting that Ca^2+^ mainly serves to support PKC activation.

Although all pathways of canonical signaling (cAMP, Ca^2+^ and PKC) contributed to P2Y_11_/IL-1R-mediated CCL20 production (Fig. 9A), Epac1 was found to regulate the response. CCL20 secretion stimulated by ATPγS plus IL-1 was strongly enhanced by Epac1 inhibitor (R)-CE3F4 (≈100% increase) (Fig. 9B). However, in contrast to sTNFR2 release, the rolipram-boosted CCL20 secretion could no longer be increased by Epac1 inhibition.

## Discussion

**Fig. 10:**
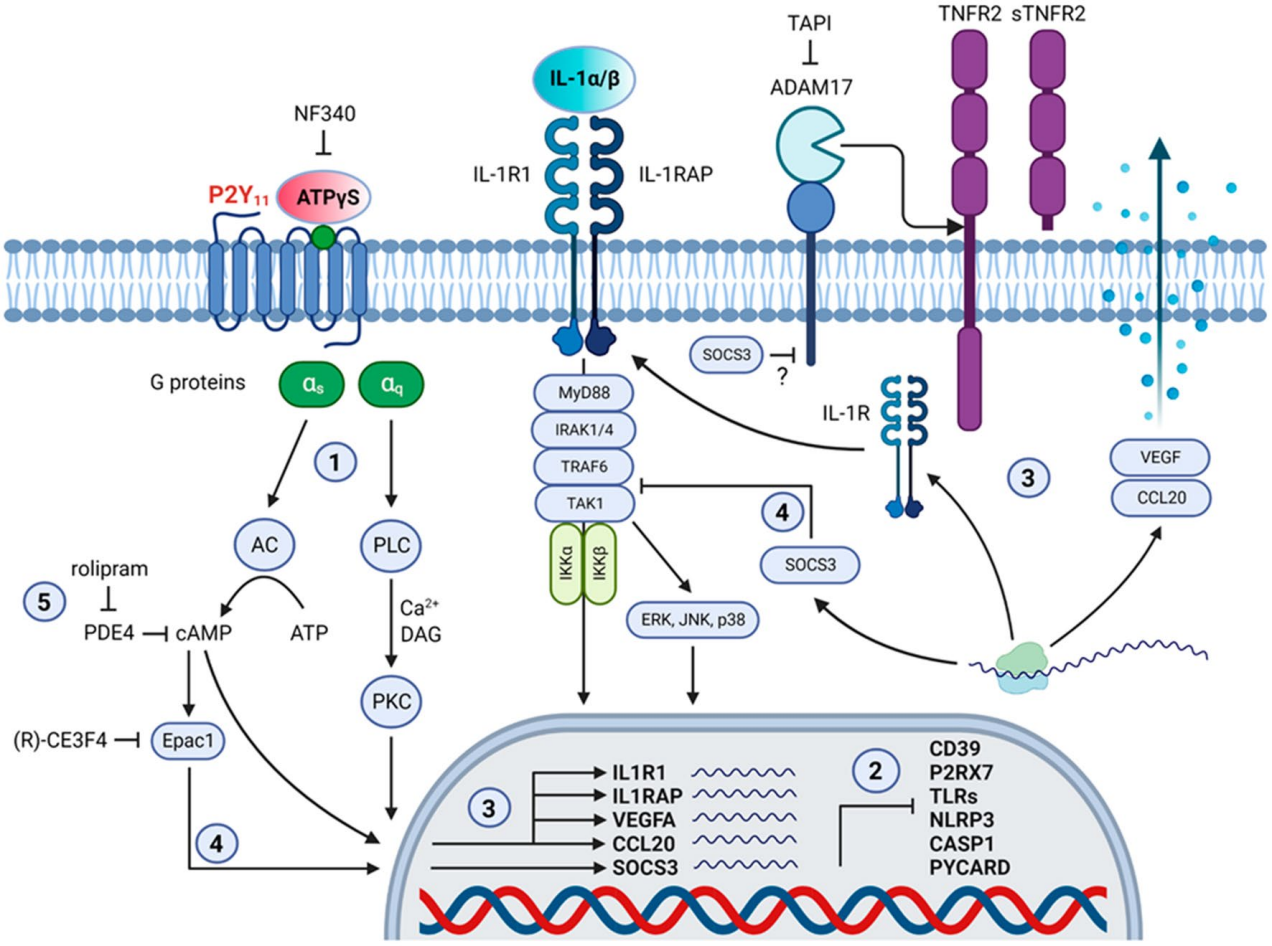
Graphical summary of P2Y_11_ anti-inflammatory and pro-angiogenic signaling in human M2 macrophages.** 1** Canonical signaling: the ATP receptor P2Y_11_ couples to PLC (via G_q_) to induce Ca^2+^ and PKC signaling as well as to AC (via G_s_) to induce cAMP signaling. **2** P2Y_11_ signaling is self-sustaining as it causes the downregulation of both, the ecto-ATPase CD39 (ENTPD1) and the pro-inflammatory ATP receptor P2X_7_. Anti-inflammatory effects of P2Y_11_ signaling include the deactivation of genes encoding TLRs and inflammasome components (NLRP3, CASP1, PYCARD). **3** P2Y_11_/IL-1R crosstalk: P2Y_11_-induced upregulation of IL-1R renders M2 macrophages highly sensitive to the effects of exogenous IL-1 cytokines, which enhance the P2Y_11_-driven secretory response (VEGF, CCL20) by activation of the IL-1R signaling cascade. P2Y_11_/IL-1R crosstalk also causes the ADAM17-mediated release of sTNFR2. **4** Strong or excessive cAMP signaling can result in Epac1-dependent induction of SOCS3, which is known to attenuate IL-1R signaling through targeting of the TRAF6/TAK1 complex. Epac1 inhibitor (R)-CE3F4 enhanced the P2Y_11_/IL-1R-driven release of soluble TNFR2 (sTNFR2) and CCL20, with little effects on VEGF, suggesting that an Epac1/SOCS3 axis can control P2Y_11_/IL-1R signaling and ADAM17 pathways. **5** The potentiation of most P2Y_11_ effects by rolipram-induced PDE4 inhibition emphasizes the importance of cAMP signaling in the P2Y_11_-mediated polarization of anti-inflammatory and pro-angiogenic M2 macrophages

We, and others, have previously shown that human P2Y_11_ is upregulated during M-CSF driven monocyte-to-macrophage differentiation [8, 12]. P2Y_11_ equips these cells with the ability to translate the extracellular danger signal ATP into cytoprotective responses. For this purpose, P2Y_11_ engages cAMP, Ca^2+^ and PKC signaling to promote IL-1R dependent anti-inflammatory and pro-angiogenic responses in human M2 macrophages (Fig. 10). We previously demonstrated that P2Y_11_ anti-inflammatory signaling includes the release of soluble TNF receptors as well as the suppression of LPS-induced TNF-α secretion [15]. Pro-angiogenic effects of P2Y_11_ could also be detected but were limited to CXCL8 (IL-8) secretion [8, 10, 15].

To confirm and extend the concept of P2Y_11_/IL-1R anti-inflammatory and pro-angiogenic signaling, we have now performed the first NanoString-based transcriptome analysis of native P2Y_11_ activation in its natural environment as a starting point and then validated the regulation of promising candidate genes at the protein level. In accordance with our previous observation in the astrocytoma-based recombinant cell system [15], P2Y_11_ activation in primary human monocyte-derived macrophages resulted in strong upregulation of IL-1R mRNA and protein. Moreover, IL1RAP was also upregulated. IL1RAP, also known as IL-1R3, encodes the IL-1R accessory protein, which functions as a co-receptor of the ligand-binding IL1R1 in IL-1 signaling. This observation was surprising, because IL1RAP is normally not regulated [23], with the exception of certain myeloid malignancies, where upregulated IL1RAP is known to correlate with poor prognosis [42]. Given the enhanced expression of P2Y_11_ in myeloid cells [8, 14], upregulation of IL1RAP in these hematopoietic tumors may in fact be driven by P2Y_11_ signaling.

Although IL-1R was upregulated in response to P2Y_11_ activation, gene and cell surface expression of the ecto-ATPase CD39 were downregulated, revealing a useful mechanism that ensures stabilization of extracellular ATP, the natural P2Y_11_ agonist, and thus facilitates prolonged anti-inflammatory P2Y_11_ signaling. The anti-inflammatory signature of P2Y_11_ activation that emerged from the NanoString analysis was impressive and included downregulation of the pro-inflammatory, pro-apoptotic ATP receptor P2X_7_ as well as of TLR5, TLR7 and TLR8. Moreover, in line with the current view that GPCRs can modulate NLRP3 inflammasome activation [43], P2Y_11_ activation also caused downregulation of all three NLRP3 inflammasome components (NLRP3, CASP1, PYCARD).

An inevitable consequence of P2Y_11_-induced inflammasome deactivation in M2 macrophages is their inability to secrete bioactive IL-1ß. The concomitant P2Y_11_-driven upregulation of IL-1R, on the other hand, renders P2Y_11_-activated M2 macrophages highly sensitive to exogenous IL-1 cytokines, which cause IL-1R activation and MyD88-dependent signaling ultimately leading to the induction of TGFß-activated kinase 1 (TAK1), IkB kinase (IKK) or mitogen-activated protein kinases (MAPK) (ERK, JNK, p38) (Fig. 10) [23, 44]. In this manner, IL-1R can support P2Y_11_, which has previously been shown to engage IKK and ERK as critical signaling components [8]. P2Y_11_-driven reprogramming thus enables M2 macrophages to respond efficiently to IL-1 produced during the pro-inflammatory phase by initiating an anti-inflammatory process that finally facilitates the resolution of inflammation.

The anti-inflammatory and pro-angiogenic responses triggered by P2Y_11_ also depended on Ca^2+^ and cAMP. The crosstalk between the second messengers Ca^2+^ and cAMP is well documented [45]. Our observation that Ca^2+^ chelation using BAPTA-AM suppressed ATPγS-induced and rolipram-enhanced responses to a similar degree suggested that the rolipram-induced increase in intracellular cAMP may serve to support Ca^2+^ signaling. cAMP activates PKA, which further promotes Ca^2+^ release from internal stores by directly phosphorylating voltage-gated Ca^2+^ channels [45]. The resulting high concentrations of Ca^2+^ in turn stimulate Ca^2+^-dependent ACs. P2Y_11_, which is the only P2Y receptor that couples to G_q_ (Ca^2+^) and G_s_ (cAMP), is perfectly equipped to initiate such mutual enhancement of Ca^2+^ and cAMP signaling. The resulting sustained levels of cAMP trigger translocation of the PKA catalytic subunit to the nucleus, where it supports the expression of anti-inflammatory genes [17].

The expression of SOCS proteins is induced or enhanced by pro-inflammatory cytokines, including IL-1ß, as part of a classical negative-feedback loop. In our study, the expression of SOCS3, a major regulator of inflammation [46], was upregulated during P2Y_11_ signaling and strongly enhanced by cAMP. This is in accordance with a previous report that had identified SOCS3 as a target of the cAMP effector Epac1 [18], and with the finding that SOCS3 can antagonize the effects of cAMP [47]. Altogether, these observations suggested that an Epac1-SOCS3 axis regulates cAMP signaling induced by P2Y_11_. In line with this consideration, Epac1 inhibitor (R)-CE3F4 further enhanced P2Y_11_-driven and ADAM17-mediated sTNFR2 release resulting in even higher levels of sTNFR2. In addition, Epac1 inhibition further increased P2Y_11_/IL-1R-induced CCL20 secretion. Our results thus establish a cAMP surveillance function of Epac1 in P2Y_11_ signaling and represent an example of ADAM17 regulation by Epac1, which has not been reported before. Epac1 has previously been implicated in P2X_7_/P2Y_11_ signaling in tumor-derived vascular endothelium, because pharmacological Epac1 activation reproduced effects that were observed after P2 receptor activation [48]. However, this work did not clarify whether increased cAMP levels resulted from AC activation through P2Y_11_-activated G_s_ proteins or through P2X_7_-mobilized Ca^2+^. In addition, NF157 was used to imply P2Y_11_ involvement. In contrast to NF340, NF157 is not fully selective among P2 receptors and has limited potency at the P2Y_11_ receptor [10, 13].

The exact targets of the Epac1/SOCS3-mediated control of P2Y_11_ signaling have not yet been identified. However, SOCS3, which is also activated by IL-1, has been shown to inhibit IL-1 signaling as part of a negative-feedback regulation by targeting the TRAF6/TAK1 complex [25]. Specifically, SOCS3 inhibited the IL-1 induced IKK/NF-κB and JNK/p38 pathways by binding and inactivating TRAF6. In addition, cAMP has been shown to inhibit ERK via Epac1-dependent induction of SOCS3 [49], altogether suggesting that P2Y_11_/IL-1R anti-inflammatory signaling is itself limited by a cAMP/Epac1/SOCS3-axis, which dampens IKK/NF-κB and MAPK pathways (Fig. 8). Moreover, P2Y_11_-induced IL-1R2 (IL1R2) and IL-1R antagonist (IL1RN) may contribute to the self-limitation of P2Y_11_/IL-1R signaling.

In addition to the anti-inflammatory effects, P2Y_11_ signaling caused an angiogenic switch. We, and others, previously reported IL8 (CXCL8) as a target of P2Y_11_ signaling [8, 10, 15]. Here, we identified the pro-angiogenic factors VEGF [50] and CCL20 [51] as additional P2Y_11_ targets. Consistent with the P2Y_11_/IL-1R crosstalk described in the present study, both VEGF and CCL20 are known to be induced by IL-1 [52, 53]. Compared to VEGF, CCL20 secretion was more strictly controlled. Although CCL20 mRNA was strongly increased in response to P2Y_11_ stimulation and further enhanced in the presence of rolipram, CCL20 protein could not be detected. IL-1 cytokines were obviously required to induce CCL20 mRNA translation and CCL20 secretion. In line with the work from Dinarello’s lab [38, 54], P2Y_11_ may serve as the first signal that rapidly activates the genes encoding IL-1 and IL-1R as well as CCL20. Cells containing untranslated or poorly translated mRNAs are now primed and small amounts of a second signal may immediately trigger mRNA translation. IL-1 is known to act as a second signal and to enhance its own expression [38], explaining our current observation that recombinant IL-1 can enhance all P2Y_11_-induced secretory responses. However, in accordance with our kinetic analyses, the particularly strong responses that are induced by P2Y_11_/cAMP-mediated IL-1R activation and enhanced by exogenous IL-1 are likely to depend on prolonged signaling and de novo or enhanced gene expression.

Our NanoString-based mRNA expression analysis revealed that human monocyte-derived macrophages generated with M-CSF did not express CCR6, the sole receptor for CCL20, indicating that the P2Y_11_-induced release of CCL20 represents a form of paracrine signaling.

The CCL20/CCR6 axis has been shown to promote cancer progression through pro-angiogenic and immunosuppressive effects as well as through direct stimulation of cancer cell migration and proliferation [55]. CCL20 may be pro-angiogenic by inducing invasion, sprouting and migration of CCR6-expressing endothelial cells [51]. CCL20 produced in the tumor microenvironment may also selectively attract Th17 cells expressing high levels of CCR6 [56]. This CCR6^+^ Th subset has been implicated in the IL-17-mediated induction of pro-angiogenic factors in the tumor microenvironment [57, 58]. Finally, the CCL20/CCR6 axis may also be immunosuppressive by inducing the selective recruitment of FoxP3-positive regulatory T cells [59]. Taken together, CCL20 secreted from P2Y_11_-activated M2 macrophages may directly promote tumor growth or indirectly support tumor progression through the recruitment of regulatory and pro-angiogenic immune cells.

CCL20 has also been implicated in inflammatory processes including inflammatory diseases [55], raising the question of whether P2Y_11_/IL-1R-driven CCL20 production is pro- or anti-inflammatory. Although we have no clear answer yet, it is important to note that ATPγS in combination with IL-1 induced only low levels of CCL20. An increase in intracellular cAMP, which is mainly considered to be anti-inflammatory [17], was required to enable robust CCL20 secretion, suggesting that P2Y_11_/IL-1R-induced CCL20 serves homeostatic purposes.

In summary, our work in human M2 macrophages provides strong evidence that the G protein-coupled ATP receptor P2Y_11_ develops its cyto- and tissue-protective effects through crosstalk with IL-1R (Fig. 10). P2Y_11_ engages all components of canonical signaling (Ca^2+^, PKC and cAMP) to stimulate anti-inflammatory and pro-angiogenic responses. P2Y_11_ has great potential for therapeutic development. From a translational point of view, targeting of P2Y_11_ may be desirable in inflammatory and infectious diseases as well as in malignant disorders. Although agonistic P2Y_11_ targeting would be required to attenuate macrophage-mediated hyper-inflammation as for instance associated with Covid-19 [60], antagonistic targeting would be necessary for an effective therapy of cancer. Myeloid malignancies with elevated IL1RAP [42] and CCR6^+^ cancers that respond to CCL20 such as liver cancer [55] might be first candidates.

## Supplementary Information

Below is the link to the electronic supplementary material.

Supplementary file1 (PDF 924 KB)

## Data Availability

All data generated or analyzed during this study are included in this published article. Additional transcriptome profiling data are also available on request from the corresponding author [MT]. Some data may not be made available because of privacy or ethical restrictions.

## References

[1] Carta S, Penco F, Lavieri R, Martini A, Dinarello CA, Gattorno M (2015). Cell stress increases ATP release in NLRP3 inflammasome-mediated autoinflammatory diseases, resulting in cytokine imbalance. Proc Natl Acad Sci USA.

[2] Rubartelli A, Lotze MT, Latz E, Manfredi A (2013). Mechanisms of sterile inflammation. Front Immunol.

[3] Junger WG (2011). Immune cell regulation by autocrine purinergic signalling. Nat Rev Immunol.

[4] Di Virgilio F, Sarti AC, Coutinho-Silva R (2020). Purinergic signaling, DAMPs, and inflammation. Am J Physiol Cell Physiol.

[5] Idzko M, Ferrari D, Eltzschig HK (2014). Nucleotide signalling during inflammation. Nature.

[6] Klaver D, Thurnher M (2021). Control of macrophage inflammation by P2Y purinergic receptors. Cells.

[7] Jacobson KA, Delicado EG, Gachet C, Kennedy C, von Kugelgen I, Li B (2020). Update of P2Y receptor pharmacology: IUPHAR review 27. Br J Pharmacol.

[8] Gruenbacher G, Gander H, Rahm A, Dobler G, Drasche A, Troppmair J (2019). The human G protein-coupled ATP receptor P2Y11 is associated with IL-10 driven macrophage differentiation. Front Immunol.

[9] Marteau F, Gonzalez NS, Communi D, Goldman M, Boeynaems JM, Communi D (2005). Thrombospondin-1 and indoleamine 2,3-dioxygenase are major targets of extracellular ATP in human dendritic cells. Blood.

[10] Meis S, Hamacher A, Hongwiset D, Marzian C, Wiese M, Eckstein N (2010). NF546 [4,4'-(carbonylbis(imino-3,1-phenylene-carbonylimino-3,1-(4-methyl-phenylene)-car bonylimino))-bis(1,3-xylene-alpha, alpha'-diphosphonic acid) tetrasodium salt] is a non-nucleotide P2Y11 agonist and stimulates release of interleukin-8 from human monocyte-derived dendritic cells. J Pharmacol Exp Ther.

[11] Ledderose C, Bromberger S, Slubowski CJ, Sueyoshi K, Aytan D, Shen Y (2020). The purinergic receptor P2Y11 choreographs the polarization, mitochondrial metabolism, and migration of T lymphocytes. Sci Signal.

[12] Layhadi JA, Fountain SJ (2019). ATP-evoked intracellular Ca(2+) responses in M-CSF differentiated human monocyte-derived macrophage are mediated by P2X4 and P2Y11 receptor activation. Int J Mol Sci.

[13] Dreisig K, Kornum BR (2016). A critical look at the function of the P2Y11 receptor. Purinergic Signal.

[14] Kennedy C (2017). P2Y11 receptors: properties, distribution and functions. Adv Exp Med Biol.

[15] Gruenbacher G, Gander H, Dobler G, Rahm A, Klaver D, Thurnher M (2021). The human G protein-coupled ATP receptor P2Y11 is a target for anti-inflammatory strategies. Br J Pharmacol.

[16] Communi D, Govaerts C, Parmentier M, Boeynaems JM (1997). Cloning of a human purinergic P2Y receptor coupled to phospholipase C and adenylyl cyclase. J Biol Chem.

[17] Gerlo S, Kooijman R, Beck IM, Kolmus K, Spooren A, Haegeman G (2011). Cyclic AMP: a selective modulator of NF-kappaB action. Cell Mol Life Sci.

[18] Sands WA, Woolson HD, Milne GR, Rutherford C, Palmer TM (2006). Exchange protein activated by cyclic AMP (Epac)-mediated induction of suppressor of cytokine signaling 3 (SOCS-3) in vascular endothelial cells. Mol Cell Biol.

[19] Wiejak J, van Basten B, Hamilton G, Yarwood SJ (2019). Genome-wide mapping defines a role for C/EBPbeta and c-Jun in non-canonical cyclic AMP signalling. Cells.

[20] von Kugelgen I (2021). Molecular pharmacology of P2Y receptor subtypes. Biochem Pharmacol.

[21] Muller CE, Namasivayam V (2021). Recommended tool compounds and drugs for blocking P2X and P2Y receptors. Purinergic Signal.

[22] Kelly JJ, Barnes PJ, Giembycz MA (1996). Phosphodiesterase 4 in macrophages: relationship between cAMP accumulation, suppression of cAMP hydrolysis and inhibition of [3H]R-(-)-rolipram binding by selective inhibitors. Biochem J.

[23] Boraschi D, Italiani P, Weil S, Martin MU (2018). The family of the interleukin-1 receptors. Immunol Rev.

[24] Gross O, Thomas CJ, Guarda G, Tschopp J (2011). The inflammasome: an integrated view. Immunol Rev.

[25] Frobose H, Ronn SG, Heding PE, Mendoza H, Cohen P, Mandrup-Poulsen T (2006). Suppressor of cytokine Signaling-3 inhibits interleukin-1 signaling by targeting the TRAF-6/TAK1 complex. Mol Endocrinol.

[26] Robson SC, Wu Y, Sun X, Knosalla C, Dwyer K, Enjyoji K (2005). Ectonucleotidases of CD39 family modulate vascular inflammation and thrombosis in transplantation. Semin Thromb Hemost.

[27] Harding SD, Armstrong JF, Faccenda E, Southan C, Alexander SPH, Davenport AP (2022). The IUPHAR/BPS guide to PHARMACOLOGY in 2022: curating pharmacology for COVID-19, malaria and antibacterials. Nucleic Acids Res.

[28] Schachter JB, Harden TK (1997). An examination of deoxyadenosine 5'(alpha-thio)triphosphate as a ligand to define P2Y receptors and its selectivity as a low potency partial agonist of the P2Y1 receptor. Br J Pharmacol.

[29] Marteau F, Le Poul E, Communi D, Communi D, Labouret C, Savi P (2003). Pharmacological characterization of the human P2Y13 receptor. Mol Pharmacol.

[30] Cattaneo M, Lecchi A, Ohno M, Joshi BV, Besada P, Tchilibon S (2004). Antiaggregatory activity in human platelets of potent antagonists of the P2Y 1 receptor. Biochem Pharmacol.

[31] Kim YC, Lee JS, Sak K, Marteau F, Mamedova L, Boeynaems JM (2005). Synthesis of pyridoxal phosphate derivatives with antagonist activity at the P2Y13 receptor. Biochem Pharmacol.

[32] Seifert R, Lushington GH, Mou TC, Gille A, Sprang SR (2012). Inhibitors of membranous adenylyl cyclases. Trends Pharmacol Sci.

[33] Zumerle S, Cali B, Munari F, Angioni R, Di Virgilio F, Molon B (2019). Intercellular calcium signaling induced by ATP potentiates macrophage phagocytosis. Cell Rep.

[34] Jovanovic DV, Di Battista JA, Martel-Pelletier J, Jolicoeur FC, He Y, Zhang M (1998). IL-17 stimulates the production and expression of proinflammatory cytokines, IL-beta and TNF-alpha, by human macrophages. J Immunol.

[35] Akahoshi T, Oppenheim JJ, Matsushima K (1988). Interleukin 1 stimulates its own receptor expression on human fibroblasts through the endogenous production of prostaglandin(s). J Clin Invest.

[36] Matsushima K, Oppenheim JJ (1985). Calcium ionophore (A23187) increases interleukin 1 (IL-1) production by human peripheral blood monocytes and interacts synergistically with IL-1 to augment concanavalin A stimulated thymocyte proliferation. Cell Immunol.

[37] Spriggs MK, Lioubin PJ, Slack J, Dower SK, Jonas U, Cosman D (1990). Induction of an interleukin-1 receptor (IL-1R) on monocytic cells. Evidence that the receptor is not encoded by a T cell-type IL-1R mRNA. J Biol Chem.

[38] Dinarello CA (1991). Interleukin-1 and interleukin-1 antagonism. Blood.

[39] Swendeman S, Mendelson K, Weskamp G, Horiuchi K, Deutsch U, Scherle P (2008). VEGF-A stimulates ADAM17-dependent shedding of VEGFR2 and crosstalk between VEGFR2 and ERK signaling. Circ Res.

[40] Courilleau D, Bouyssou P, Fischmeister R, Lezoualc'h F, Blondeau JP (2013). The (R)-enantiomer of CE3F4 is a preferential inhibitor of human exchange protein directly activated by cyclic AMP isoform 1 (Epac1). Biochem Biophys Res Commun.

[41] Lin TJ, Maher LH, Gomi K, McCurdy JD, Garduno R, Marshall JS (2003). Selective early production of CCL20, or macrophage inflammatory protein 3alpha, by human mast cells in response to Pseudomonas aeruginosa. Infect Immun.

[42] Barreyro L, Will B, Bartholdy B, Zhou L, Todorova TI, Stanley RF (2012). Overexpression of IL-1 receptor accessory protein in stem and progenitor cells and outcome correlation in AML and MDS. Blood.

[43] Tang T, Gong T, Jiang W, Zhou R (2018). GPCRs in NLRP3 inflammasome activation, regulation, and therapeutics. Trends Pharmacol Sci.

[44] Dunne A, O'Neill LA (2003). The interleukin-1 receptor/Toll-like receptor superfamily: signal transduction during inflammation and host defense. Sci STKE.

[45] Borodinsky LN, Spitzer NC (2006). Second messenger pas de deux: the coordinated dance between calcium and cAMP. Sci STKE.

[46] Carow B, Rottenberg ME (2014). SOCS3, a major regulator of infection and inflammation. Front Immunol.

[47] Bellezza I, Neuwirt H, Nemes C, Cavarretta IT, Puhr M, Steiner H (2006). Suppressor of cytokine signaling-3 antagonizes cAMP effects on proliferation and apoptosis and is expressed in human prostate cancer. Am J Pathol.

[48] Avanzato D, Genova T, Fiorio Pla A, Bernardini M, Bianco S, Bussolati B (2016). Activation of P2X7 and P2Y11 purinergic receptors inhibits migration and normalizes tumor-derived endothelial cells via cAMP signaling. Sci Rep.

[49] Woolson HD, Thomson VS, Rutherford C, Yarwood SJ, Palmer TM (2009). Selective inhibition of cytokine-activated extracellular signal-regulated kinase by cyclic AMP via Epac1-dependent induction of suppressor of cytokine signalling-3. Cell Signal.

[50] Thomas JL, Eichmann A (2013). The power of VEGF (vascular endothelial growth factor) family molecules. Cell Mol Life Sci.

[51] Benkheil M, Van Haele M, Roskams T, Laporte M, Noppen S, Abbasi K (2018). CCL20, a direct-acting pro-angiogenic chemokine induced by hepatitis C virus (HCV): potential role in HCV-related liver cancer. Exp Cell Res.

[52] Brand OJ, Somanath S, Moermans C, Yanagisawa H, Hashimoto M, Cambier S (2015). Transforming growth factor-beta and interleukin-1beta signaling pathways converge on the chemokine CCL20 promoter. J Biol Chem.

[53] Tanaka T, Kanai H, Sekiguchi K, Aihara Y, Yokoyama T, Arai M (2000). Induction of VEGF gene transcription by IL-1 beta is mediated through stress-activated MAP kinases and Sp1 sites in cardiac myocytes. J Mol Cell Cardiol.

[54] Schindler R, Clark BD, Dinarello CA (1990). Dissociation between interleukin-1 beta mRNA and protein synthesis in human peripheral blood mononuclear cells. J Biol Chem.

[55] Kadomoto S, Izumi K, Mizokami A (2020). The CCL20-CCR6 axis in cancer progression. Int J Mol Sci.

[56] Yu Q, Lou XM, He Y (2015). Preferential recruitment of Th17 cells to cervical cancer via CCR6-CCL20 pathway. PLoS ONE.

[57] Ye J, Livergood RS, Peng G (2013). The role and regulation of human Th17 cells in tumor immunity. Am J Pathol.

[58] Numasaki M, Fukushi J, Ono M, Narula SK, Zavodny PJ, Kudo T (2003). Interleukin-17 promotes angiogenesis and tumor growth. Blood.

[59] Chen KJ, Lin SZ, Zhou L, Xie HY, Zhou WH, Taki-Eldin A (2011). Selective recruitment of regulatory T cell through CCR6-CCL20 in hepatocellular carcinoma fosters tumor progression and predicts poor prognosis. PLoS ONE.

[60] Merad M, Martin JC (2020). Pathological inflammation in patients with COVID-19: a key role for monocytes and macrophages. Nat Rev Immunol.

