# Supplementary material for: The P2Y11 receptor of human M2 macrophages activates canonical and IL-1 receptor signaling to translate the extracellular danger signal ATP into anti-inflammatory and pro-angiogenic responses
Source: Cell Mol Life Sci. 2022 Sep 15;79(10):519. doi: 10.1007/s00018-022-04548-z (PMC9476423; doi:10.1007/s00018-022-04548-z)
Supplement: Supplementary file 1 — Supplementary file1 (PDF 924 KB) [file 18_2022_4548_MOESM1_ESM.pdf]

Supplementary Information (SI)

The P2Y$_{11}$ receptor of human M2 macrophages activates canonical and IL-1 receptor signaling to translate the extracellular danger signal ATP into anti-inflammatory and pro-angiogenic responses

Cellular and Molecular Life Sciences

Dominik Klaver[1] · Hubert Gander[1] · Gabriele Dobler[1] · Andrea Rahm[1] · Martin Thurnher[1]

[1]Immunotherapy Unit, Department of Urology, Medical University of Innsbruck, 6020 Innsbruck,

Austria – EUROPE; martin.thurnher@i-med.ac.at

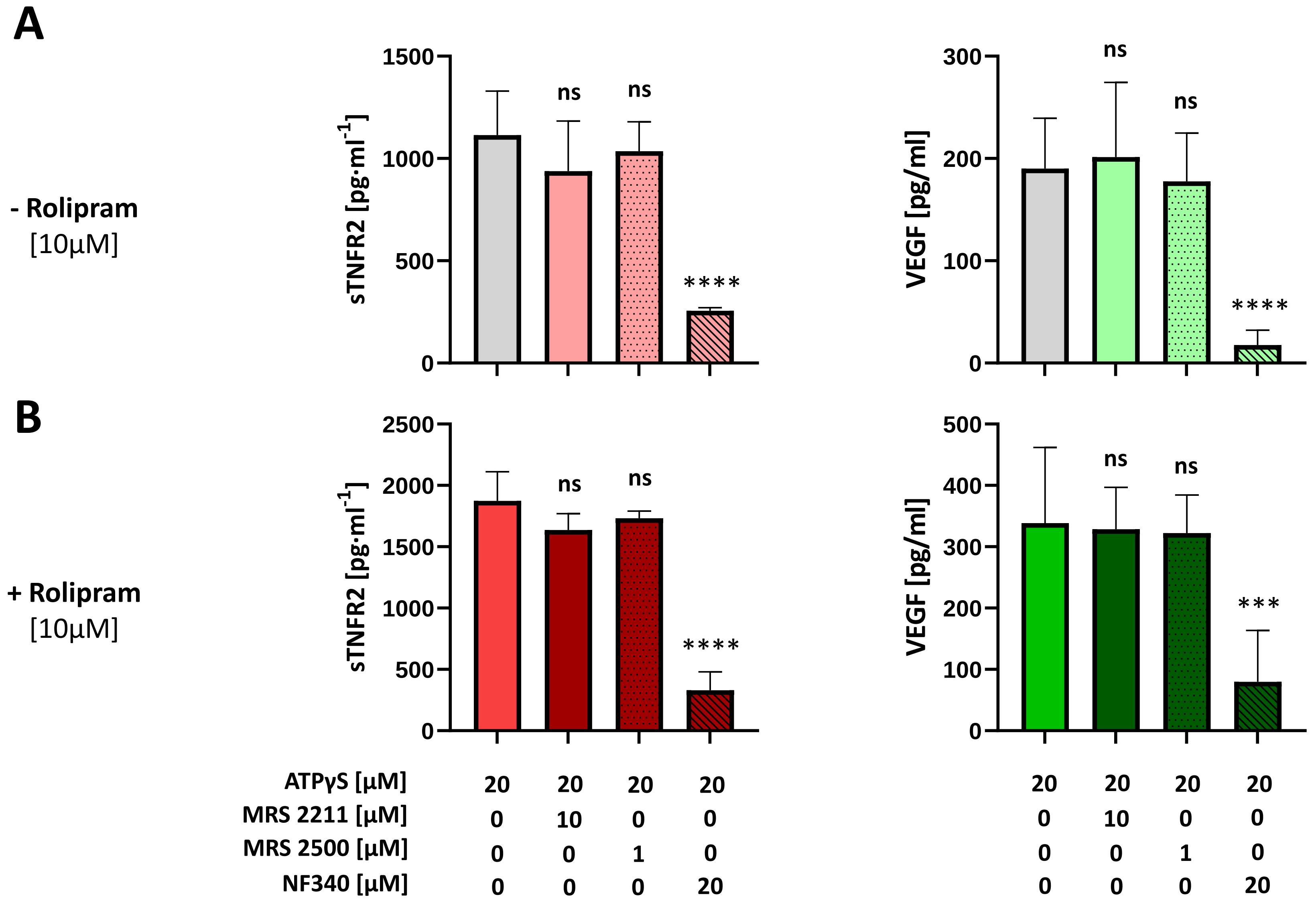

Figure S1: sTNFR2 release and VEGF secretion in response to $P2Y_{11}$ activation is not influenced by $P2Y_1$ or $P2Y_{13}$ receptors. M2 macrophages were treated for 24 h with the $P2Y_{11}$ receptor agonist ATPγS (20 µM) in the absence (**A**) or presence (**B**) of the PDE4-selective inhibitor rolipram (10 µM). sTNFR2 and VEGF levels were measured in cell culture supernatants. The $P2Y_{11}$-specific antagonist NF340 (20 µM) as well as the $P2Y_1$- and $P2Y_{13}$-specific antagonists MRS 2500 (1 µM) and MRS 2211 (10 µM), respectively, were used to confirm that ATPγS-mediated responses were specific to $P2Y_{11}$ receptor stimulation (n=3). ns, not significant; ***$p < 0.001$, ****$p < 0.0001$.

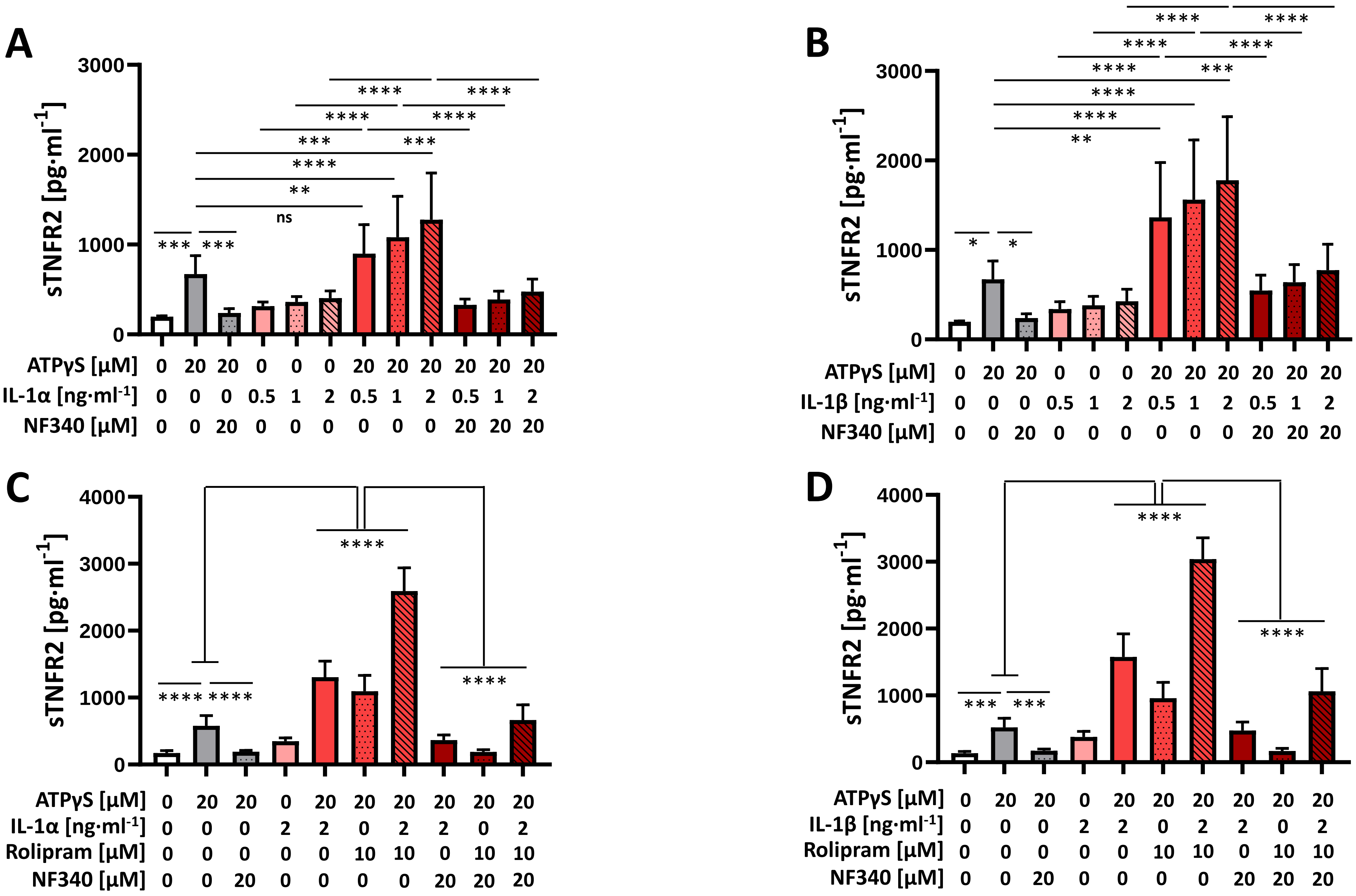

Figure S2: $P2Y_{11}$ activation on human M2 macrophages increases IL-1 responsiveness and enhances sTNFR2 release, which is even more pronounced when intracellular cAMP levels are raised through PDE inhibition. M2 macrophages were treated for 24 h with increasing doses of IL-1α (**A, C**) or IL-1β (**B, D**), either alone or in combination with the $P2Y_{11}$ receptor agonist ATPγS (20 µM), in the presence or absence of the PDE4-selective inhibitor rolipram (10 µM) (**C, D**). sTNFR2 levels were measured in cell culture supernatants. NF340 (20 µM) was used to confirm that agonist-mediated responses were specific to $P2Y_{11}$ receptor stimulation (n = 3). *$p < 0.05$, **$p < 0.01$, ***$p < 0.001$, ****$p < 0.0001$.

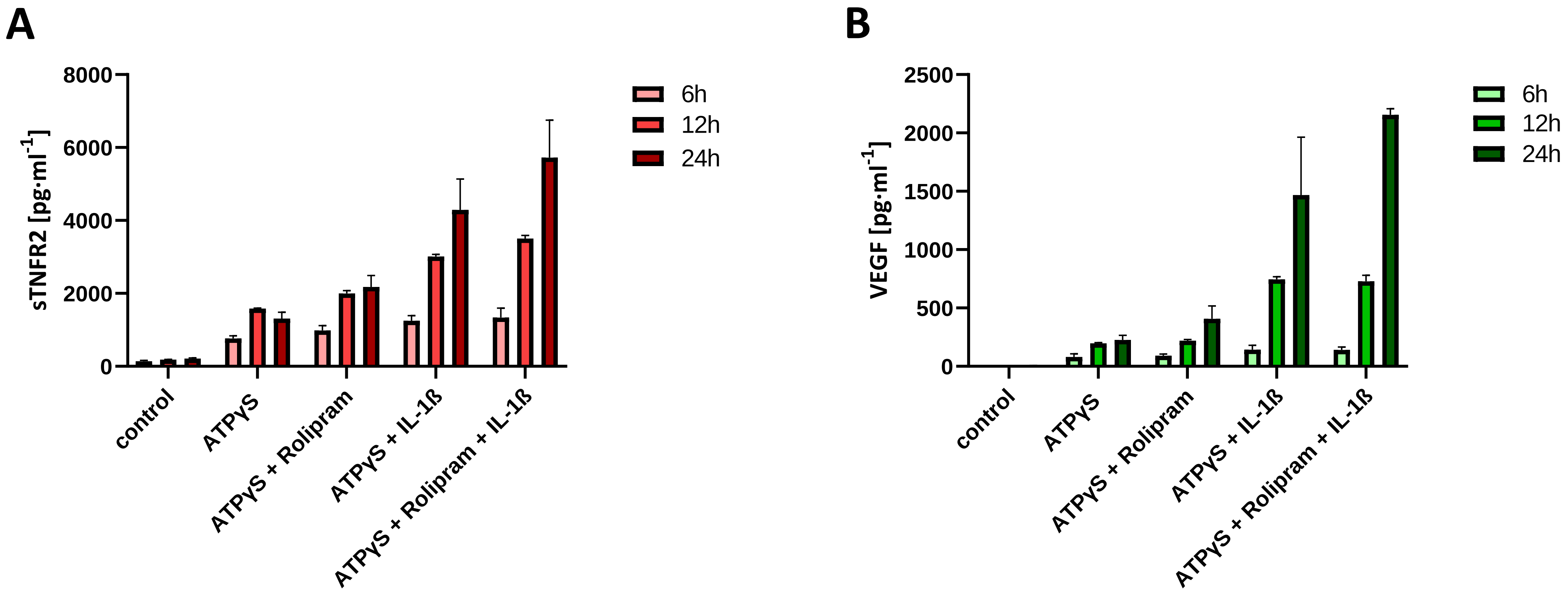

Figure S3: Time course of sTNFR2 release and VEGF secretion upon $P2Y_{11}$ activation on human M2 macrophages. M2 macrophages were treated for 6 h, 12 h, or 24 h with the $P2Y_{11}$ agonist ATPγS (20 µM), either alone or in combination with IL-1β (2 ng·ml^{-1}), in the presence or absence of the PDE4-selective inhibitor rolipram (10 µM). sTNFR2 (**A**) and VEGF (**B**) levels were measured in cell culture supernatants (n=3).

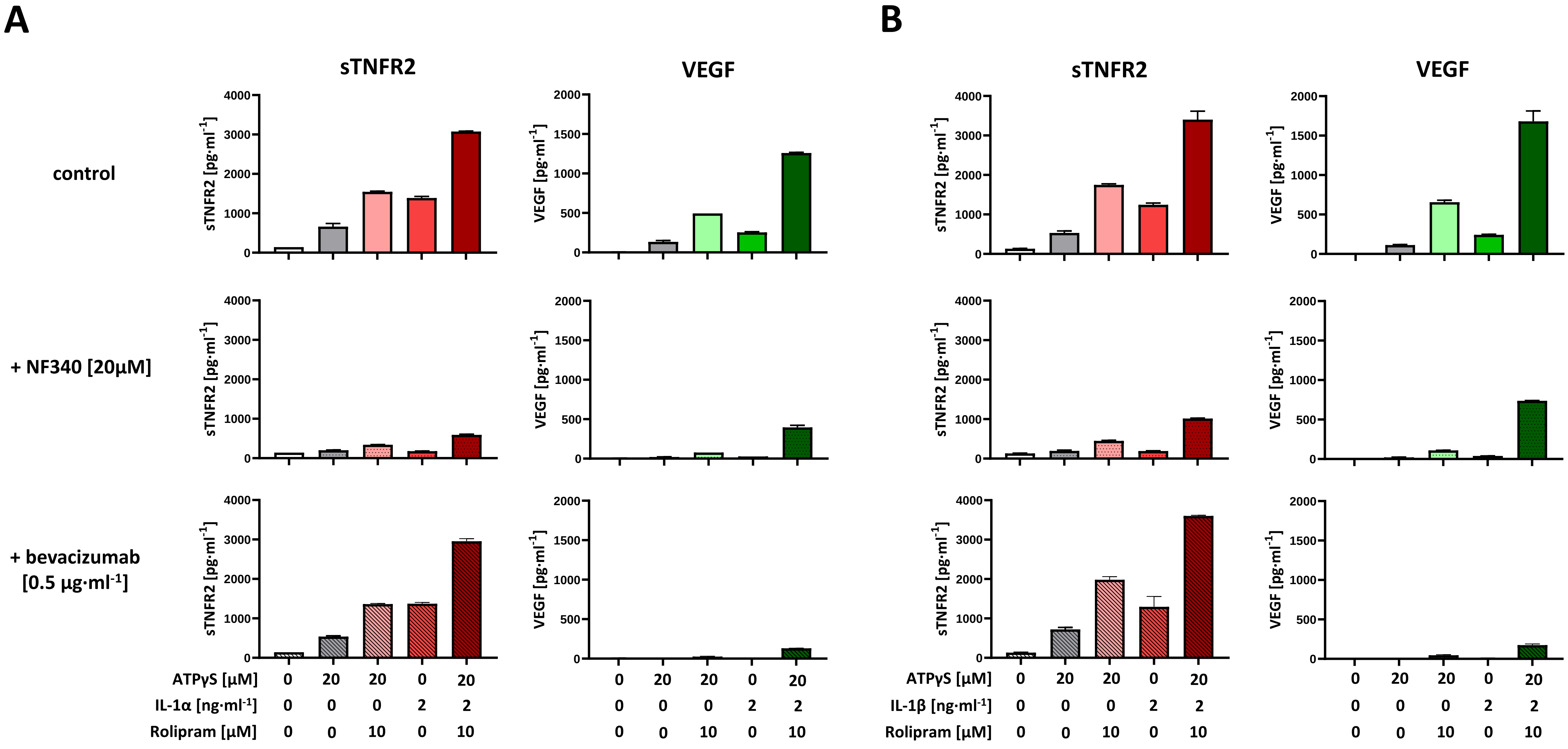

Figure S4: VEGF secretion in response to $P2Y_{11}$ activation has no effect on the shedding of sTNFR2. M2 macrophages were treated for 24 h with the $P2Y_{11}$ receptor agonist ATPγS (20 µM) either alone or in combination with IL-1α (2 ng·ml^{-1}) (**A**) or IL-1β (2 ng·ml^{-1}) (**B**), in the presence or absence of the PDE4-selective inhibitor rolipram (10 µM) (**A, B**). The humanized anti-VEGF monoclonal antibody bevacizumab (0,5 µg·ml^{-1}) (**A, B** bottom row) was utilized in order to neutralize exogenous VEGF. sTNFR2 and VEGF levels were measured in cell culture supernatants. The antagonist NF340 (20 µM) was used to confirm that agonist-mediated responses were specific to $P2Y_{11}$ receptor stimulation (**A, B** middle row).

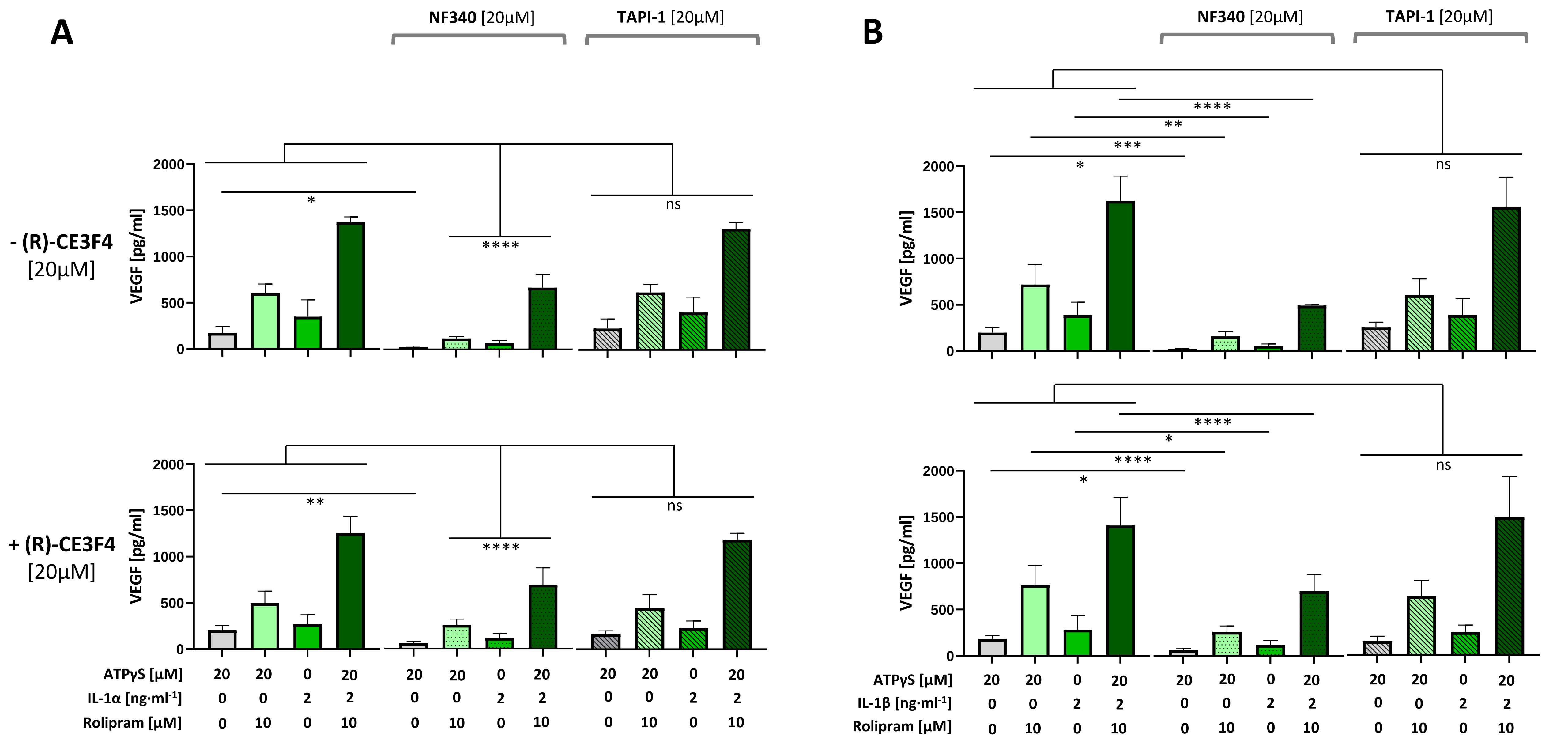

Figure S5. The P2Y$_{11}$ receptor driven secretion of VEGF is not controlled by Epac1 and does not depend on TACE/ADAM17. M2 macrophages were treated for 24 h with the P2Y$_{11}$ receptor agonist ATPγS (20 μM) either alone or in combination with the selective Epac1 inhibitor (R)-CE3F4 (20 μM), in the presence or absence of IL-1α (2 ng·ml^{-1}) (**A**) or IL-1β (2 ng·ml^{-1}) (**B**) as well as with or without the PDE4-selective inhibitor rolipram (10 μM) (**A, B**). The TACE/ADAM17 inhibitor TAPI-1 (20 μM) was used to clarify whether TACE/ADAM17 is involved in the shedding of VEGF (**A, B**). VEGF levels were measured in cell culture supernatants. The antagonist NF340 (20 μM) was used to confirm that agonist-mediated responses were specific to P2Y$_{11}$ receptor stimulation (n = 3). ns, not significant; $*p < 0.05$, $**p < 0.01$, $***p < 0.001$, $****p < 0.0001$.
